# A Novel Tetrameric PilZ Domain Structure from Xanthomonads

**DOI:** 10.1371/journal.pone.0022036

**Published:** 2011-07-07

**Authors:** Tso-Ning Li, Ko-Hsin Chin, Kit-Man Fung, Ming-Te Yang, Andrew H.-J. Wang, Shan-Ho Chou

**Affiliations:** 1 Institute of Biochemistry, National Chung-Hsing University, Taichung, Taiwan, Republic of China; 2 National Chung-Hsing University Biotechnology Center, National Chung-Hsing University, Taichung, Taiwan, Republic of China; 3 Institute of Molecular Biology, National Chung-Hsing University, Taichung, Taiwan, Republic of China; 4 Institute of Biological Chemistry, Academia Sinica, Nankang, Taipei, Taiwan, Republic of China; 5 Graduate Institute of Basic Medical Science, China Medical University, Taichung, Taiwan, Republic of China; Griffith University, Australia

## Abstract

PilZ domain is one of the key receptors for the newly discovered secondary messenger molecule cyclic di-GMP (c-di-GMP). To date, several monomeric PilZ domain proteins have been identified. Some exhibit strong c-di-GMP binding activity, while others have barely detectable c-di-GMP binding activity and require an accessory protein such as FimX to indirectly respond to the c-di-GMP signal. We now report a novel tetrameric PilZ domain structure of XCC6012 from the plant pathogen *Xanthomonas campestris* pv. *campestris* (*Xcc*). It is one of the four PilZ domain proteins essential for *Xcc* pathogenicity. Although the monomer adopts a structure similar to those of the PilZ domains with very weak c-di-GMP binding activity, it is nevertheless interrupted in the middle by two extra long helices. Four XCC6012 proteins are thus self-assembled into a tetramer via the extra heptad repeat α3 helices to form a parallel four-stranded coiled-coil, which is further enclosed by two sets of inclined α2 and α4 helices. We further generated a series of XCC6012 variants and measured the unfolding temperatures and oligomeric states in order to investigate the nature of this novel tetramer. Discovery of this new PilZ domain architecture increases the complexity of c-di-GMP-mediated regulation.

## Introduction

Recent studies have identified c-di-GMP as a universal secondary messenger molecule that is extensively involved in regulating bacterial pathogenicity [1]–[9]. Even though the synthesis and degradation of c-di-GMP have been extensively studied, little is known about the downstream effector mechanism carried out by c-di-GMP currently. PilZ domain [10] was first found to be the receptor for c-di-GMP via a bioinformatics study [4]. In fact, many PilZ-domain containing proteins from a variety of different bacteria have been found to bind to c-di-GMP with variable affinities, ranging from sub-µM to µM. These proteins include PlzD [7] and VCA0042 from *V. cholerae* [6], YcgR from *E. coli* and *G. xylinus* [11], DgrA from *C. crescentus* [12], and PA4608 [5] and Alg44 from *P. aeruginosa* [13]. In these proteins, two highly conserved regions for c-di-GMP binding were found, namely, the RxxxR and D/NxSxxG motifs. However, due to the highly diverse functions exhibited by c-di-GMP, it is possible that receptors with different or degenerate c-di-GMP binding motifs other than the canonical PilZ domain may exist. For example, PelD, which mediates the production of polysaccharide biosynthesis from *P. aeruginosa*, contains only the RxxxR motif but not the D/NxSxxG motif [14], while FleQ, which regulates the flagella biosynthesis genes in *P. aeruginosa*, lacks both motifs completely [9]. Recently, a Clp (c-AMP receptor protein like protein) from the plant pathogen *Xcc* was found to be a moderately strong c-di-GMP binder [15]–[17] that is responsible for controlling the expression of approximately 300 downstream genes [18]. However, it also lacks the RxxxR and D/NxSxxG motifs present in the canonical PilZ domain [16]. In addition, a different (c-di-GMP)_2_ binding site comprised mainly of the RxxD motif in the GGDEF domain was also found to be involved in controlling the cellular c-di-GMP concentration via an allosteric product inhibition mechanism [19]. Thus it appears that more alternative c-di-GMP binding motifs will be revealed in further study.

The coiled-coil proteins are remarkable for the diversity of conformations they can adopt [20], [21]. Typically, several α-helices are wrapped around each other to form a left-handed supercoil [22]–[25]. The coiled-coil proteins are prevalent in biology and are predicted to be present in approximately 10% of all eukaryotic proteins with widely ranging functions [26], [27]. The coiled-coil protein features a seven residue periodicity (heptad repeat, usually annotated as **a–b–c–d–e–f–g**), with hydrophobic residues occurring at the first (**a**) and fourth (**d**) positions to form the coiled-coil, and charged residues at the **e** and **g** positions forming extra interhelical electrostatic interactions to determine the orientation and specificity between the helices [22]–[25]. Finally, the remaining three positions (**b**, **c**, and **f**) are normally occupied by hydrophilic residues and are exposed to the surrounding solvent. However, these rules only serve as a rough guide for governing the interactions between left-handed dimeric coiled coils. Subtle alternations of amino acids at the **a**, **d**, **e**, and **g** positions could change the oligomerization [28], orientation [29], specificity [22], handedness [30], and offset [31], [32] of coiled coils that are required for achieving various distinct functions. After fifty years, the research about coiled-coils is still intensive [20], [21], [23], [26], [33]–[36].

**Figure 1:**
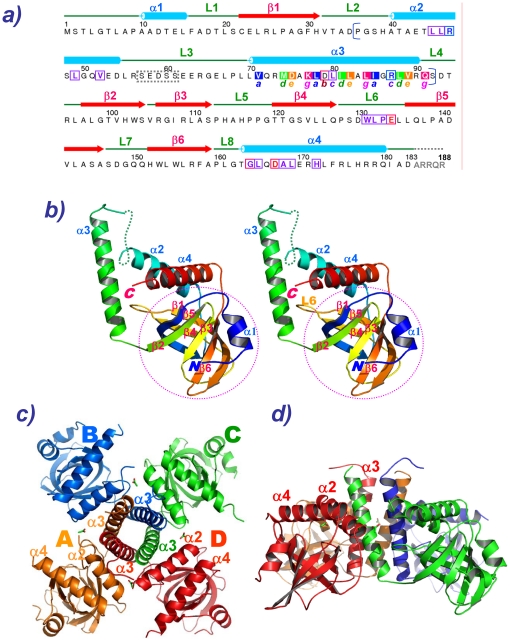
Sequence and Structure of XCC6012. a) Sequence of XCC6012 with associated secondary structural elements. The sample was prepared by the LIC method and contains an extra SNA sequence at the N-terminal end after the TEV cleavage. The full-length XCC6012 plus the N-terminal SNA amino acids were employed for crystallization. Since the N-terminal SNA sequence, several central α2-α3 loop sequence, and the C-terminal ARRQR sequences remain invisible in the electron density map, they were either omitted (for the SNA sequence), boxed (for the central α2-α3 loop) or colored in gray (for the ARRQR sequence). Helix α3 contains three turns of heptad repeats, with residues in position **a** highlighted in blue, position **d** highlighted in green, position **e** highlighted in orange, and position **g** highlighted in red. Unlike other simple tetrameric coiled coils, the residues from the α2 helix and the L6 loop, as well as the residues from positions **e**, **g**, **b**, and **c**, are also involved in forming a type II interaction network, while residues in helices α2 and α4 are involved in forming type III interactions. Residues participating in forming type II and type III hydrophobic interactions were boxed in purple, while residues participating in forming ion pairs were boxed in blue and red for the positively-charged residues and negatively-charged residues, respectively. b) Cartoon stereo picture of the monomeric XCC6012 tertiary structure color-coded from blue (N-terminus) to red (C-terminus). The helices and strands were annotated in blue and red letters, respectively. c) Cartoon top-down view of the tetrameric XCC6012 structure. Four XCC6012 proteins form a symmetric tetramer correlated by a four-fold axis. d) Side view of the “house-like” architecture of the tetrameric XCC6012 structure. The central “pillar” domain contains the four α3 helices, the “roof-top” domain contains the α2/α4 helices, and the “corner-stone” domain contains the four PilZ domains.

The *Xcc* strain 8004 genome contains four PilZ genes, and each of these genes was found to play a role in causing the *Xcc* pathogenicity [37]. Similarly, *Xcc* strain 17 genome also contains four PilZ genes (Li et al, unpublished result). Intriguingly, while three PilZ genes were found to adopt an intact PilZ domain sequence, *xcc6012* from *Xcc* strain 17, which is completely identical to *xc*2249 from *Xcc* strain 8004 (GenBank access number: **AAY49304.1**, see Supplementary Fig. S1), contains an insert in the middle by a long sequence of 57 amino acids (bracketed in Fig. 1 a). Thus we determined to solve the structure and function of this peculiar PilZ domain protein. Interestingly, after solving the XCC6012 crystal structure, we found that it adopts a novel quaternary PilZ variant that contains an intact PilZ-like domain, yet is interrupted in the middle by two long helices (α2 and α3). Four such XCC6012 molecules are intertwined into a stable tetramer via the heptad repeat α3 helices that form a parallel four-stranded coiled-coil. Unlike other regular tetrameric coiled-coils, that of XCC6012 is further enclosed by two sets of α2 and α4 helices in the outer surfaces, with most residues in the heptad repeat except those at position **f** also actively participating in forming this unusual complex helix bundle. The quaternary structure of XCC6012 is found to adopt a novel “house-like” architecture, with a central pillar domain comprising the four vertical α3 helices, a roof-top domain comprising the eight inclined α2 and α4 helices, and four corner-stone domains comprising the PilZ domain. The discovery of this unusual tetrameric PilZ domain structure shall increase the complexity of PilZ domains and their potential functions mediated by c-di-GMP.

## Results

### Tertiary Structure of the XCC6012 Monomer

The cloning, protein expression, and crystallization of XCC6012 have been well documented [38], [39]. In brief, Se-Met substituted XCC6012 was found to form square-shaped crystals belonging to the space group P2_1_, and contain four monomers and four acetate molecules per asymmetric unit. The final structure was obtained by repeated refinement to a resolution of 2.1 Å, with values of 22.0% and 24.6% for the R_cryst_ and R_free_, respectively. The data collection and refinement statistics are listed in Table 1. The overall geometry of XCC6012 is quite good, with the vast majority of backbone torsional angles (90.5%) located in the most favorable region of the Ramanchandran plot. The reminders of the backbone torsional angles are located in the additionally allowed region (9.1%) or in the generously allowed region (0.4%). No residue is located in the disallowed region. The final experimental electron density map reveals the positions of most amino acid residues of the tetramer in the asymmetric unit except for the five residues from Ser57 to Ser 61 in chain A, the eleven residues from Leu55 to Gly65 in chain B, the six residues from Arg56 to Ser 61 in chain C, and the thirteen residues from Leu55 to leu67 in chain D, which are located in the L3 region and remain invisible even after repeated refinement.

**Table 1:** Statistics of data collection and structural refinement of Se-Met Xc6012.

Beamline	NSRRC BL13B1		
	Inflection^b^	High remote^b^	Peak^b^
Wavelength (Å)	0.97910	0.96388	0.97884
Space group	P2_1_	P2_1_	P2_1_
Unit cell parameters	a = 58.84, b = 89.37, c = 87.35, β = 105.4	a = 58.84, b = 89.37, c = 87.35, β = 105.4	a = 58.84, b = 89.37, c = 87.35, β = 105.4
Resolution range (Å)	30–2.11 (2.19–2.11)*	30–2.1 (2.18–2.1)*	30–2.2 (2.28–2.2)*
Unique observations	49875 (4940)*	50523 (5020)*	44136 (4415)*
Redundancy	3.8 (3.4)*	3.9 (3.4)*	7.7 (7.0)*
Completeness (%)	99.4 (98.6)*	99.5 (99.0)*	99.7 (99.8)*
R_merge_ (%)	5.6 (43.8)*	3.9 (45.4)*	5.5 (43.2)*
*I*/*σ(I)*	20.0 (2.8)*	21.2 (3.4)*	32.8 (4.5)*
R_free_ test set size (%)		5%	
**Refinement statistics**			
R_cryst_/R_free_ a (%)		22.0/24.6	
**Model content**			
Protein residues		715	
Waters		416	
Acetate		4	
**B-factors**			
Backbone atoms		45.4	
Side-chain atoms		47.5	
Acetate atoms		13.4	
Water oxygen atoms		42.7	
**Ramachandran plot** (%)			
Residues in most favorable regions		90.5	
Residues in additionally allowed regions		9.1	
Residues in generously allowed regions		0.4	
**r.m.s.d from ideal geometry**			
Bonds (Å)		0.01	
Angles (°)		1.40	

*Values in parenthesis are for the outermost shell while the preceding values refer to all data.

a *R*_free_ is the same as *R*_cryst_ but for 5.0% of the total reflections chosen at random and omitted from refinement.

b Friedel mates were considered separately as unique reflections in the calculation of these statistics.

**Figure 2:**
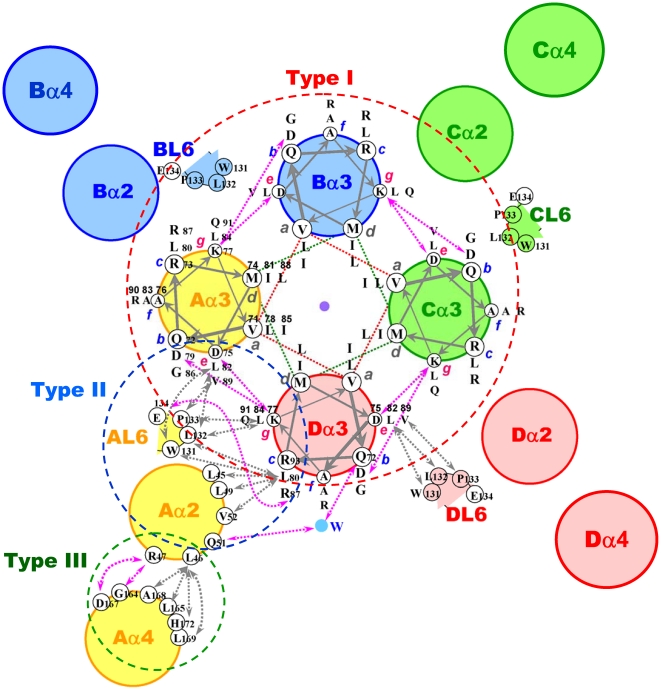
Depiction of Interaction Networks of Tetrameric XCC6012 in a Helical Wheel. Helices and loops of the four protomers are annotated as A, B, C, D, and filled with yellow, blue, green, and pink colors. Residues in each heptad position involved in the central coiled-coil interactions are labeled, with residues and numbers displayed in each position. Type I interactions are dash circled in red, type II in blue and type III in green. Only interactions between the Dα3/AL6/Aα2/Aα3 structural elements are annotated in detail. Similarly, helices α4 are involved in type III interactions, but only interactions between the Aα2/Aα4 helices are displayed in detail.

XCC6012 is composed of a six-stranded β-barrel flanked by four helices (Fig. 1a and 1b) with a secondary structure topology of α1/β1/α2/α3/β2/β3/β4/β5/β6/α4 (PDB code: 3RQA and RCSB code: rcsb065245) It adopts a typical PilZ domain structure similar to that of XCC1028 [40], yet is interrupted by two extra long helices of α2 and α3 between the β1 and β2 strands. The coil connecting the α2 and α3 helices is the most flexible region in the monomer as revealed by their higher B-factors (Fig. S6). The additional α3 helix from each monomer marginally interacts with the other structural elements of its own subunit, but is positioned to interact with the α2 and α3 helices from other flanking subunits, thus forming a stable tetrameric structure (Fig. 1c and Fig. 2).

### XCC6012 Is a Unique PilZ-Like Protein from Xanthomonads

Although XCC6012 is considered to be a PilZ-like protein [37], it is nevertheless an orphan protein in the xanthomonads, because no conserved homologue with a sequence similarity higher than 20% outside this genera could be detected after a NCBI BLASTp search (http://blast.ncbi.nlm.nih.gov/Blast.cgi?PROGRAM=blastp&BLAST_PROGRAMS=blast). However, four proteins with a very good sequence identity higher than 80% with XCC6012 from the xanthomonads were discovered (Fig. S1). Using the DALI program [41] (http://ekhidna.biocenter.helsinki.fi/dali_server/), a structural homologue search for the determined XCC6012 structure via comparison of the entire structure against the PDB returned several PilZ-domain structures with Z-scores of 8.1, 7.8, 7.6, 5.9 and 5.5 for the 1YLN, 2RED, 2GJG, 1YWU and 3DSG structures, respectively. R.m.s.d. values between these PilZ-domain structures are moderately good (2.6, 2.7, 2.6, 2.6 and 2.6 Å, respectively), and the β3-β6 strand core regions were also well superimposed, although the N-terminus and the C-terminus exhibited larger variations (see supplementary Figs. S2 and S3). Sequence identities between XCC6012 and the aforementioned PilZ-domain sequences are quite low, with values of only 14%, 13%, 13%, 15% and 19% (Fig. S2). In addition, XCC6012 contains only a partial c-di-GMP binding signature motif (SxxG, Fig. S2) and a long sequence of 57 amino acids comprising two α-helices (α2 and α3) after the β1 strand (Fig. S2). Taken together, these results indicate that XCC6012 is a sequence-unique protein from xanthomonads, and is distinctive in the PilZ domain structures.

### The Novel Quaternary Structure of XCC6012

As shown in Fig. 1a and 2, the inserted helix α3 is found to possess a heptad repeat sequence comprising three turns, with four such α3 helices self-assembling to form a parallel coiled-coil. But unlike other regular four-stranded coiled-coils, XCC6012 is further surrounded by four additional α2 and α4 helices, forming an intricate 12-helix bundle architecture (Fig. 1c and 2). Three types of interactions are observed in this unique coiled-coil (Fig. 2): Type I consisting of parallel α3 coiled-coil interactions (circled in red in Fig. 2); type II consisting of interconnected helix-helix (α2­α3) and helix-loop interactions (L6-α3, circled in blue Fig. 2), and type III consisting of α2-α4 helical interactions (circled in green in Fig. 2).

#### Type I Parallel Coiled-Coil Interactions

**Figure 3:**
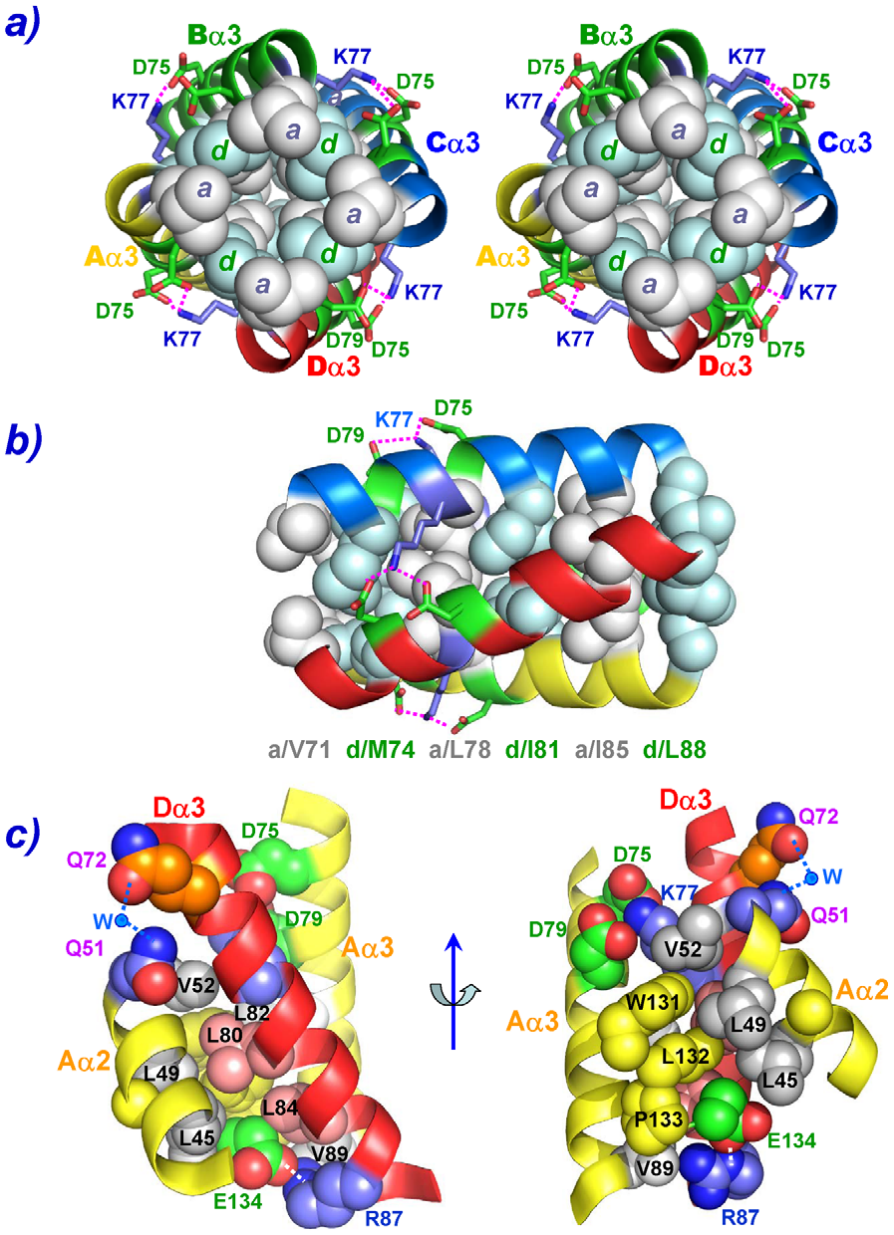
Space-filling Cartoon Pictures of Inter-Domain Interactions Involved in Forming Type I and Type II Interaction Networks. a) Type I coiled-coil interactions are drawn in the top-down view in stereo. Residues from position **a** and **d** of helices α3 form alternative interdigitated hydrophobic layers. Residues participating in hydrophobic interactions are color-coded in either gray spheres for layer **a** or in light-blue spheres for layer **d**, while ion pairs between the Lys77 and the Asp75/Asp79 residues are represented as sticks and connected by dotted pink lines. b) Similar type I coiled-coil interactions drawn in horizontal orientation. The six layers of hydrophobic interaction networks are apparent from this orientation; c) Type II interactions from residues located at the outer surfaces of α3 with residues at the inner surfaces of α2 and loop L6 residues drawn in two different perspectives. Hydrophobic residues from helix Aα3 and Aα2 are drawn in gray spheres, while those from Dα3 are drawn in light pink spheres, and hydrophobic residues from loop L6 are drawn in yellow spheres. The carbons of the positively-charged residues are drawn in light blue spheres, while those of the negatively-charged residues in green spheres. The polar oxygen and nitrogen atoms are drawn in red and blue spheres, respectively.

Type I interactions result from the assemblage of four individual heptad repeat α3 helices into a well-known parallel coiled-coil, except that each α3 helix is located at a position interacting with α2 helix from an adjacent protomer (Fig. 1c). The XCC6012 tetramer is related by a four-fold symmetrical axis parallel to the α3 helices. As shown in Fig. 2, the heptad repeat of the α3 helix contains purely the hydrophobic amino acids Val71, Leu78, Ile85 situating at position **a**, and Met74, Ile81, Leu88 at position **d**, respectively. These residues are interdigitated between the **a** and **d** layers and form excellent hydrophobic interactions (Fig. 3a and 3b). Due to tight hydrophobic packing, no room is available for water or metal ions to fit. In the surface of the coiled-coil, a pair of salt bridges between residue Lys77 at position **g** and residues Asp75 and Asp79 at position **e** and **b**, respectively, are observed (Fig. 3a and 3b), which stabilize the coiled-coil as are generally observed in other helix bundles [22], [42]. In addition, several hydrophobic residues are found to locate at positions **c** (Leu80), **e** (Leu82, Val89), or **g** (Leu84) that are normally occupied by hydrophilic residues and exposed to solvent (Fig. 2) [28], [31], [32]. These altered hydrophobic residues are responsible for forming additional type II helical interactions as described below.

#### Type II Helical-Helical α3-α2 and Helical-Loop α3-L6 Interaction

Except for the regular hydrophobic and charged interactions occurring in a normal coiled-coil domain, some unique interactions for amino acid residues located at positions **c**, **e**, and **g** of the helical wheel are also observed in the tetrameric XCC6012. These antiparallel Dα3-Aα2 type inter-promoter interactions (Fig. 2), along with the helical-loop interactions of the Dα3-AL6 and Aα3-AL6 types (Fig. 2), form the second set of interactions in this novel helix bundle (Figs. 3c). These unique interactions further enhance the tetramer stability and specificity of XCC6012. As shown in Fig. 2, residues Leu80 and Leu84 in the Dα3 are extensively involved in interacting with the Leu45/Leu49/Val52 hydrophobic residues in the Aα2 helix and the β carbon of Glu134 in the AL6 loop, as well as with the Trp131/Leu132/Pro133 hydrophobic residues in the AL6 loop, respectively (Fig. 2, marked in dotted gray arrows and Fig. 3), while residues Leu82 and Val89 in the Aα3 helix are found to interact with the Trp131/Leu132/Pro133 hydrophobic residues in the AL6 loop (Fig. 2, marked in dotted gray arrows and Fig. 3c). Further polar interactions are also observed in this region, such as a salt bridge between the charged residue Arg87 at position **c** of the Dα3 helix and the charged residue Glu134 in the AL6 loop (marked by dotted red arrows), as well as two H-bonds between the charged residue Gln72 at position **b** with the charged residue Gln51 in the Aα2 loop via a water molecule (Fig. 2, marked by a blue circle and Fig. 3c). These interactions are illustrated from two different perspectives in Fig. 3c. From the figure, it is clear that the hydrophobic residue Leu80 is involved in interacting with hydrophobic residues from the Aα2 helix and the AL6 loop, while the hydrophobic residues Leu82 and Val89 are involved in interacting with residues from the AL6 loop. Two indirect H-bonds between residues Gln51 and Gln72 via a water molecule and two salt bridges between the charged residues Arg87 and Glu134 (Fig. 3c) are also observed in the top and bottom segments of helix α3, respectively, enhancing the type II interactions. These results demonstrate the extensive hydrophobic and polar interaction network that is involved in this type II interaction.

#### Type III Parallel Inter-Helical Interaction

Type III α2-α4 inter-helical interactions are not as extensive as those in the type I or type II interaction network, but nonetheless also involve some hydrophobic and polar interactions as shown in Fig. 2 and Fig. S4. Residue Leu46 in the Aα2 loop interacts extensively with residues Leu165/Ala168/Leu169/His172 in the Aα4 helix, while residue Arg47 in the Aα2 helix interacts with the side chain oxygen atoms of Asp167 and the main chain atom of Gly164 in the Aα4 helix.

Overall, extensive hydrophobic and polar interactions are detected in this unique tetrameric coiled-coil, including sixteen hydrophobic interactions and eight salt bridges in the type I network, forty hydrophobic interactions, four salt bridges, and eight H-bonds in the type II network and sixteen hydrophobic interactions, four salt bridges, and four H-bonds in the type III network.

We calculated the amount of monomer surface buried due to the subunit contact in this tetramerirc PilZ domain structure by using the Protein-Protein interface comparison server (http://www.bioinformatics.sussex.ac.uk/protorp/) [43]. When only two subunits were calculated, we found that approximately 11% or 1185 Å^2^ of interface accessible surface area was buried. Besides, a considerable percentage of interface is contributed by the non-polar residues (64%), indicating a strong association of the monomer in this dimeric form. However, it is important to note that each heptad repeat in helix α3 in fact interacts with three other α3s to form a four-helical bundle, not just one as calculated using only two subunits. Therefore, the monomer surface buried for each monomer in the tetramer should be much more than that calculated from the program.

We also plotted the tetrameric XCC6012 structure in both the B-factor and electrostatic formats (Fig. S6) to determine if there is any potential region for interaction with other ligands or proteins. The B-factor plot (top of Fig. S6) indicates that except for the terminal ends, the α2-α3 regions have the highest B-factors. In the van der Waals plot of (bottom of Fig. S6), there are four surface patches that are enriched with positively-charged amino acid residues (marked by green squares). These results indicate that XCC6012 may possibly interact with other ligands or proteins through the top “roof” region of this new house architecture (Fig. S1c). Without further experimental data, it is difficult to truly determine the protein surface region of interaction. However, it is interesting to note that the neighborhood genes of *xcc6012* are those involved in the flagellar-promoted bacterial motion (upstream, colored in green) or in the bacterial two component signal transduction process (downstream, colored in yellow). The next step, which is currently underway, is to use the ITC method to detect whether these neighborhood gene products can interact with XCC6012.

### Biophysical Studies of the XCC6012 Variants

**Figure 4:**
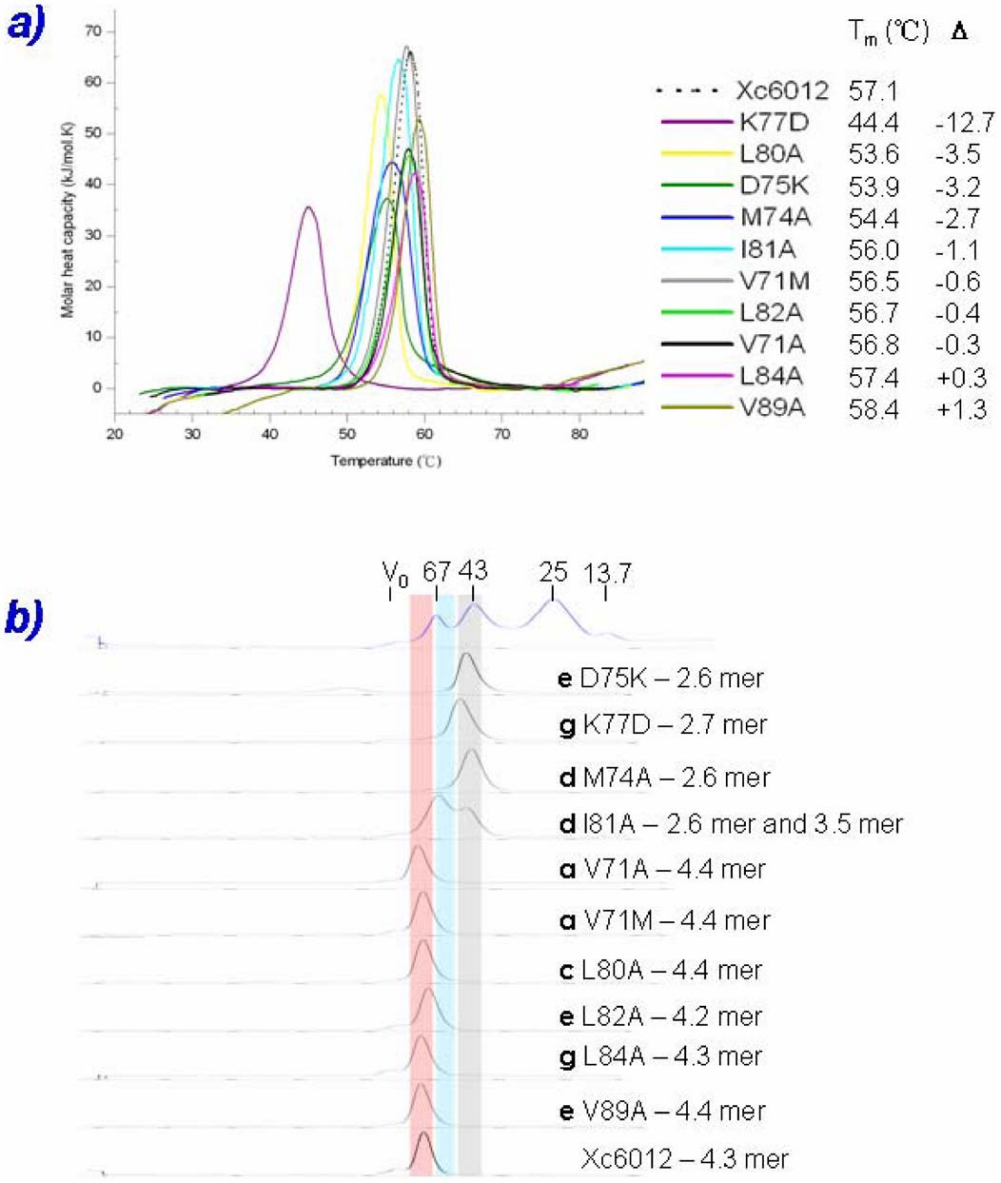
Biophysical Studies of the Wild Type and Altered XCC6012 at a Concentration of 38 µM. a) Unfolding temperatures recorded by the DSC measurements. Their melting temperatures and temperature differences to that of the wild type are also listed in the figure inset; b) Oligomerization states measured by size-exclusion chromatography. The oligomer values calculated from the data are also listed in the figure inset. The MW standards with known Stokes radii are shown at the top as a control.

To gain more insights into the origin of the stability of this novel tetrameric PilZ domain structure, we constructed a series of XCC6012 variants on key amino acids involved in the above described type I and type II interactions and carried out the unfolding and oligomerization studies with the use of differential scanning calorimetry (DSC), size exclusion chromatography, and analytical ultracentrifugation, respectively. In the DSC studies (Fig. 4a), each XCC6012 variant was slowly heated over a range of temperatures. Heating each protein sample initially produced a slightly increasing baseline, but as heating progresses, heat is absorbed by the protein, giving rise to an endothermic peak characteristic of that protein. Once unfolding was complete, heat absorption decreased and returned to the baseline. The transition midpoints of the XCC6012 variants thus obtained and the differences to the wild type are listed in the insert of Fig. 4a. While wild type XCC6012 exhibited an unfolding temperature of 57.1°C, those of the K77D and D75K variants revealed unfolding temperature at 44.4C° and 53.9C°, which are substantially lower than those of the wild type by 12.7C° and 3.2C°, respectively. This data indicates that the electrostatic interactions between amino acid residues at heptad repeat positions **g** (K77) and **e** (D75) are crucial for stabilizing the parallel tetrameric coiled-coil, and changes of complementarily charged amino acid pairs to similarly charged amino acids cause a great destabilization for XCC6012. The reason why K77D variant exhibits a much more dramatic decrease in unfolding temperature than that of the D75K variant may possibly be due to the fact that Lys77 forms two salt bridges with Asp75 and Asp79 (Fig. 3a and 3b). Therefore a single change of Lys77 to a negative charged Asp will cause at least a two-fold drop in the unfolding temperature as expected. Interestingly, the L80A variant also causes a large unfolding temperature drop by 3.5C°. This result indicates that the inter-protomer and inter-helical hydrophobic interactions clustered by L80 as described in the previous section (Fig. 3c) is crucial for the stability of this novel tetrameric XCC6012 as well. We also observe a decreased drop in the unfolding temperature when the first **a** layer hydrophobic residue in the heptad repeat helix (V71 in Fig. 3b) changes to an Ala or Met (the V71A and V71M variants decrease by 0.3C° and 0.6C°, respectively). This temperature drop is less dramatic than that observed by substituting the first **d** layer residue (the M74A variant decreases by 2.7C°) or the second **d** layer residue (the I81A variant decreases by 1.1C°), possibly because hydrophobic stacking interrupted in the center causes a larger destabilization effect than the one interrupted at the beginning when the residue changes to a less hydrophobic one (Fig. 3b). We also find that amino acid changes at either position **e** (Leu82 and Val89) or **g** (Leu84) that interact with the AL6 loop causes less decrease or even increase in the unfolding temperature (Inset in Fig. 4a). This result may be explained by the fact that loop residues usually experience less structural constraints, and are therefore more adaptable. Therefore, their conformations are easier to adjust when necessary to accommodate the amino acid changes in the opposing interacting L6 helices.

In addition, we also carried out size exclusion chromatography experiments to determine the oligomerization state for each XCC6012 variant (Fig. 4b). As expected, the oligomerization states of the XCC6012 variants are roughly proportional to their unfolding temperatures; namely, the variants with lower unfolding temperature such as D75K, K77D, M74A, and I81A (with some partial trimeric state) seem to exist in a dimeric state, while variants with higher unfolding temperatures such as V71M, V71A, L82A, L84A, and V89A prefer to existing in a tetrameric state (Fig. 4b). The only exception is the L80A variant, which seems to exhibit a lower unfolding temperature, yet exists mainly in a tetrameric state. Further study is needed to explain this result.

Furthermore, we also carefully studied the oligomerization states of wild XCC6012 at two different concentrations of 6.4 µM and 38.4 µM (Fig. S7) and the oligomerization states of the V71M and K77D protein variants at 38.4 µM (Fig. S8) via the sedimentation velocity method. The data show that there is only one moving boundary for all these profiles (Fig. S7a, Fig. S7d, Fig. S8a, and Fig. S8d). We also fitted the multiple scans at different time points to a continuous size distribution with Lamm equation [44] using SEDFIT program (http://www.analyticalultracentrifugation.com), and superimposed the differences between the experimental data and the fitted curves to generate the “residuals” figures (Fig. S7b, Fig. S7e, Fig. S8b, and Fig. S8e). The minute difference values and consistent errors of residuals indicate that the fit is quite acceptable. The lower panels (Fig. S7c, Fig. S7f, Fig. S8c, and Fig. S8f) show the results of the analyses, namely, the continuous distribution of sedimentation coefficients c(s), corresponding to the data from the top moving boundary panels. The bottom panel (Fig. S7g) shows the superimposition from XCC6012 at two different concentrations. A molecular weight of 84 kDa was obtained after fitting the data with the Lamm equation, with no other species of less molecular weight observed. In Fig. S8g, the c(s) contributions from the wild type V71M and K77D variants were superimposed, which show molecular weights of the K77D and V71M variants at 39 kDa and 84 kDa, respectively. The rmsd for the entire ensemble of velocity AUC scans for XCC6012 are 0.0061 at 38.4 µM and 0.0045 at 6.4 µM, respectively. Similarly, the rmsd for the entire ensemble of velocity AUC scans are 0.0073 and 0.0061 for the V71M variant at 38.4 µM and the K77D variant at 38.4 µM, respectively. The initial frictional ratio f/f0 for fitting is set at the default value of 1.2 in the SEDFIT program for calculation. The frictional ratio is also one of the variables for the c(s) curve fitting, which converges to a weight-average value of 1.24 after fitting with the wild type protein, 1.23 with the V71M variant, and 1.27 with the K77D variant, respectively. These data clearly indicate that XCC6012 and V71M variant adopt a tetrameric structure, while the K77D variant adopts a dimeric structure. Such data are consistent with the results obtained from the gel filtration experiments (Fig. 4b). Regarding the oligomer dissociation constants, we have found that they are difficult to measure since no other species can be observed in the XCC6012 and V71M tetramer or in the K77D dimer c(s) distribution curves (Fig. S7c, Fig. S7f, Fig. S8c, and Fig. S8f). Crude estimates of their dissociation constants can, however, be obtained from the following descriptions. For a dimer↔monomer dissociation of the K77D variant, the Kd would be equal to the monomer concentration when the system is at half-saturation [45]. From Fig. S8f, if we assume that a monomer peak of nearly equal intensity can be observed, then the Kd for the K77D variant would be 6.4 µM. However, since no monomeric peak can be observed at all, the dimer form of K77D must be stable and its Kd would be smaller than 6.4 µM. Similarly for a tetramer↔monomer dissociation, the half-saturation amount of the monomer can be expressed as A_1/2_ = 2Kd^1/3^ [45], thus yielding a Kd equal to A_1/2_^3^/8 for this case. Once again assuming that a monomeric peak of nearly equal intensity can be observed, the Kd for the wild type and V71M variant would theoretically be (6.4)^3^/8 (µM)^3^ or 32 (µM)^3^. But as before, there is no monomeric peak that can be observed for the wild type XCC6012 and V71M variant, so their Kd for the tetramer would be actually much smaller than the aforementioned calculated values. These AUC experiments also indicate that XCC6012 and its protein variants form a tetramer or a dimer rapidly with small dissociation constants. Therefore only a single species can be observed in the continuous distribution of sedimentation coefficients c(s) curves. The excellent purity and stability of the tetrameric form of XCC6012 may also contribute to the formation of high quality crystals suitable for X-ray diffraction study.

### Virulence Assay of the *xcc6012* Gene

**Figure 5:**
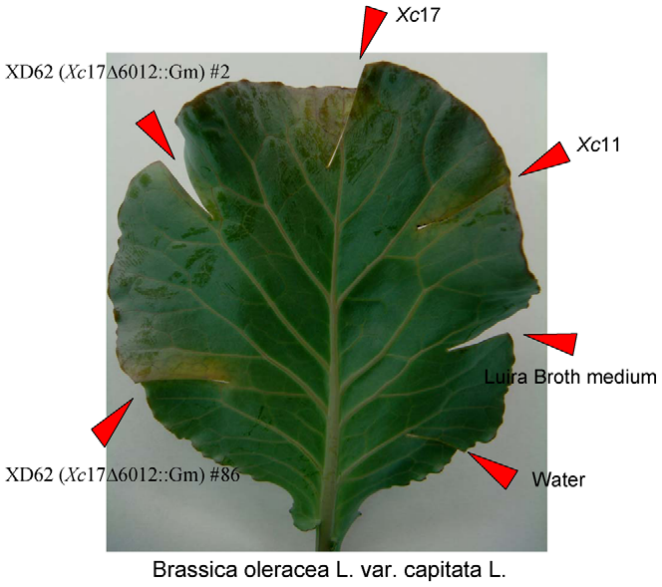
In vivo Virulence Assay for Wild type and *xcc6012* Knockout Mutants of the Xc17 Strain. Different strains and control (water and Luria Broth) were inoculated onto the cutting edges of the leaves of a potted cabbage plant. After incubating for 7 days at 26°C with an 8 hr/day lighting, the leaves were cut and photographed to show the different virulence phenotype.

*Xcc* strain 8004 contains four pathogenic *pilZ* genes, and knockout of each gene led to a variety of different disease symptoms [37]. *xc*2249 is one of the aforementioned *pilZ* genes, and its knockout was found to cause a decrease in the virulence of the Chinese radish, a reduction in the *Xcc* motility, and changes in *Xcc* extracellular enzyme production [37]. The gene sequence of *xcc6012* in the *Xcc* strain 17 is identical to that of *xc*2249 in the *Xcc* strain 8004 (Fig. S1. To check for the pathogenicity of *xcc6012* and its deletion variant toward the crucifer plant *Brassica oleracea*, we constructed an *xcc6012* knockout Xc 17 strain (XD62) through the use of a double-crossover recombination method and then introduced the knockout strain into the vascular system of cabbage by the leaf clipping method as previously described [37]. The results of this virulence assay for the wild type and *xcc6012* knockout strains are shown in Fig. 5. From the figure, one can see that the negative control of water and Luria broth medium-treated leaf clipping did not reveal any damage, yet those treated with the wild type Xc17 strain did cause a considerable disease symptoms (the less virulent Xc11 species caused a lesser degree of damage as expected). In contrast, the clipping treated with the *xcc6012* knockout strain (XD62 #2 and XD62 #86, Fig. 5) did not cause as much damage as that treated with the wild type strain. This experiment indicates that *xcc6012* is indeed a pathogenic gene. However, it is important to note that there are four PilZ genes in the *Xcc* that exhibit partially overlapping functions, and deletion of only one of these genes may not be able to diminish the *Xcc* pathogenicity completely [37].

### Comparison of XCC6012 with Other PilZ Domains

Recently, structures of several PilZ-domain proteins with conserved RxxxR and D/NxSxxG motifs were reported, including VCA0042 from *Vibrio cholerae* in either apo- (1YLN) or c-di-GMP-complexed form (2RDE) [7], PA4608 from *Pseudomonas aeruginosa* (1YWU) [5], and PP4397 from *Pseudomonas putida* (2GJG). The sequence and structural alignments of these proteins with XCC6012 are shown in Fig. S2. From the figure, it is clear that most β-strands such as β3-β6 in XCC6012 superimpose well with those in the other canonical PilZ domains, even though XCC6012 does not contain complete RxxxR and D/NxSxxG motifs, but only a partial SxxG sequence in the second motif (highlighted in red in Fig. S2). In contrast, the C-terminal end of the β1 strand and the N-terminal of the β2 strand of XCC6012 diverge to a greater extent than those of the canonical PilZ domains, due to the insertions of the extra α2 and α3 helices between the β1 and β2 strands (indicated by dotted cyan and red or green arrows in Fig. S3). Another interesting and crucial region comprises the N-terminal 15 residues, which are heavily involved in c-di-GMP binding. In XCC6012, the residues from Ala10-Leu15 form an intact helix, as do the residues of Arg123-Leu130 from PP4397 (Fig. S2). However, residues in the similar region from VCA0042 and PA4608 were found to adopt a coiled state, allowing these residues to bind to c-di-GMP with more freedom. Interestingly, helix α1 of XCC6012 is found to overlap with the c-di-GMP ligand in the ligand-bound VCA0042 complex when the two structures are superimposed (Fig S3). Before binding c-di-GMP, the N-terminal YcgR domain of VCA0042 is swung away from the c-di-GMP binding site in the C-terminal PilZ domain. However, after c-di-GMP binding, the N-terminal YcgR domain was found to rotate almost 180°, thus wrapping around the c-di-GMP ligand (Fig. S3a). Residues from both domains, including the conserved ones in the RxxxR and D/NxSxxG motifs, were found to interact with the enclosed c-di-GMP [7]. Since XCC6012 contains no intact c-di-GMP binding motifs, and its binding with c-di-GMP could not be reliably obtained by the ITC experiment (Fig. S5), it may need an additional component to respond to the c-di-GMP message. An example of such a case was recently demonstrated for the FimX_GGDEF-EAL_-PilZ_XAC1133_ complex, in which the PilZ domain of XAC1133 did not bind to c-d-GMP, but rather the message of c-di-GMP was transmitted through the c-di-GMP binding degenerate EAL domain of the FimX protein [46].

## Discussion

The coiled-coil motif is a unique system that has been extensively studied to investigate the protein folding, molecular recognition, and *de novo* protein design as described in several recent reviews [20], [23], [36]. It can adopt a variety of structures with different oligomerization, polarity, packing offset [32], and homo- versus heteromeric association [47], depending on the geometric properties of the core **a**- and **d-**layer residues as well as the outer **e-** and **g-**layer residues [42]. For example, a switch between the two-, three-, and four-stranded parallel coiled-coils in the GCN4 leucine zipper variants has been well addressed [28]. This switch was found to depend on the interior packing of residues at the **a** and **d** layers, with the presence of isoleucine at layer **a** and leucine at layer **d** forming a dimer, while the presence of leucine at layer **a** and isoleucine at layer **d** favoring a tetramer. In contrast, isoleucine at both **a** and **d** layers prefers a trimer [28]. Likewise, a detailed study on the role of interhelical **g**-**e**' ion pairs on the orientation and oligomerization of coiled-coils has also been reported [42]. However, these rules only serves as rough guides, as residues at layers **a**, **d**, **e**, and **g** all seem to play a role in determining the orientation and oligomerization of coiled-coils, and a complex interplay between these residues may exist to determine their functionality. For example, XCC6012 adopts a mixture of leucine and isoleucine at layers **a** and **d** (Fig. 2), yet it is still able to form a stable parallel tetameric coiled-coil.

Although the PilZ domain was first predicted by a bioinformatics approach to be a possible c-di-GMP receptor[4] and later experiments did prove that some PilZ domains exhibit remarkable binding affinities with c-di-GMP [5], [6], [14], it is important to note that not every PilZ domain protein binds with c-di-GMP with similar affinity. For example, for the eight PilZ orthologs in *P. aeruginosa*, seven were found to exhibit variable binding affinities with c-di-GMP. However, PA2960, after which the PilZ domain was named, did not reveal detectable c-di-GMP binding [13]. Similarly, only two PlzB and PlzD proteins out of the five PilZ orthologs in the *V. cholerae* bind c-di-GMP with considerable affinities in vitro, yet knockouts of plzB, plzD, or plzC gene resulted in significant changes in motility, biofilm formation and intestinal colonization of *V. cholerae* [6]. Thus, some PilZ domain proteins may need other accessory proteins to respond to c-di-GMP in order to exhibit their functionality. Interestingly, one PilZ domain structure of XAC1133 from *Xac* was also recently solved [46] and found to adopt an almost identical type II PilZ domain structure to that of the XCC1028 [40]. It was also demonstrated that XAC1133, which exhibits no detectable c-di-GMP binding activity, was nonetheless able to bind with PilB, an ATPase required for the type IV pilus (T4P) polymerization, and with the EAL domain of FimX, which can bind c-di-GMP and regulate the T4P biogenesis and localization in *Xac* [46]. Since FimX contains degenerate GGDEF and EAL motifs,[48]–[50] and XAC1133 also contains a degenerate PilZ motif, their interactions with the PilB protein via these degenerate motifs to control the T4P machinery via c-di-GMP is very intriguing and is worthy of further investigation.

As far as we know, our report is the first of this kind to determine a PilZ domain protein existing in a tetrameric state. In fact, XCC6012 is stable and able to form a tetramer in solution even at a low concentration of 6.4 µM, as clearly demonstrated by an analytical ultracentrifugation experiment (Fig. S7). From the results of the gel filtration and analytical ultracentrifugation experiments, it is clear that XCC6012 exists as a tetamer, but it is still inconclusive as to how XCC6012 functions in this tetrameric state. A literature search revealed several examples of regulatory proteins adopting a tetramer linked by a coiled-coil. For example, the *lac* repressor was found to comprise four N-terminal headpieces that bind to operator DNA and a C-terminal tetrameric core that binds to an inducer. The coiled-coil was found to stabilize the *lac* repressor and regulate the cooperativity of ligand binding [51], [52]. Also, the F conjugation regulatory protein TraM was found to adopt a core of parallel four-stranded coiled-coil comprising the N-terminal helices, with each C-terminal helix packing in an antiparallel arrangement around the outside of the structure [53]. Further studies are necessary to elucidate the possible roles of XCC6012 in *Xcc* pathogenicity.

## Materials and Methods

### Cloning, Expression, and Purification of Wild-type XCC6012

The *xcc6012* gene fragment was PCR-amplified from a local *Xcc* genome (*X. campestris* pv. *campestris* strain 17) with the corresponding forward and backward primers as listed in Table 2. The amplified product was cloned into a pTBSG1 vector by using the ligation-independent cloning (LIC) approach [54] to obtain the desired construct according to a previously published protocol [38]. The Se-Met XCC6012 was prepared in a similar way to that used for the native XCC6012, except that the cells were induced in a Se-Met containing M9 minimum medium until an OD_600_ of 0.8 was reached.

### Crystallization, Data Collection and Structural Refinement of Wild Type XCC6012 Crystals

The screening and optimization of XCC6012 crystallization have been published in a previous report [39]. Crystals were soaked in a cryoprotectant solution comprising the reservoir solution plus 25% (v/v) glycerol and then flash-cooled at 100 K under a stream of cold nitrogen. X-ray diffraction data were collected using the National Synchrotron Radiation Research Center (NSRRC) beamline 13B1 in Taiwan. A three-wavelength MAD data set, with 2s exposure time, 0.5° oscillation angle, and 300 mm crystal-to-film distance, was obtained up to a resolution of 2.1 Å [55]. The data were indexed and integrated using the HKL2000 processing software [56], generating data set that were 99.4% complete with overall R_merge_ of 3.9%–5.6% on intensities. The refinement of selenium atom positions (four in total), phase calculation, and density modification were carried out using the program *SnB* v2.3 [56]. Approximately 70% and 60% of the backbone and side chains of the proteins were visible from the first experimentally calculated electron density map using the Autofit function of SOLVE/RESOLVE [57]. The model obtained was manually adjusted using the XtalView/Xfit package [58]. CNS was then used for data refinement via the use of an overall B-factor refinement, followed by restrained individual B-factor refinement to a final *R*_cryst_ of 22.0% and *R*_free_ of 24.6%, respectively [59]. The crystals belong to the P2_1_ space group, and contained four XCC6012 domains per asymmetric unit. The Matthews coefficient and solvent content of the crystals are 2.67 Å^3^/Da and 54.04%, respectively. Detailed statistics about the data collection and refinement of XCC6012 crystals are summarized in Table 1.

### Construction of Single Point xcc6012 Mutants

The strains and plasmids used in constructing the *xcc6012* mutants are listed in Table 2. All single point *xcc6012* mutations were generated using the QuickChange®II Site Directed Mutagenesis Kit (Stratagene) [60], with the resulting sequences further confirmed by DNA sequencing. The forward primers (listed in Table 2) and their corresponding complementary primers were used to introduce the V71M, V71A, M74A, D75K, K77D, L80A, I81A, L82A, L84A, and V89A mutations. The protein variants were then expressed and purified as described above.

### Construction of *xcc6012* Knockout Mutants

A 0.7-kb *Nde*I/*Xho*I fragment containing the *xcc6012* gene was first digested from the pT6012 plasmid and subsequently cloned into a suicide pOK vector [61], yielding the pO6012 plasmid. The knockout of *xcc6012* was achieved by inserting a 0.8-kb *Sma*I/*Sma*I fragment containing the Gm^r^ cassette from the plasmid pUCGM [62] into the *Msc*I site at 332 nt from the start codon of the *xcc6012* gene to generate the final plasmid pO6012gm (*xcc6012*::Gm). The pO6012gm plasmid was then transformed into Xc17 by electroporation. The Kan^s^ and Gm^r^ resistant strain was selected to obtain the *xc*c6012 knockout mutant XD62 (Xc17Δ*xcc6012*::Gm).

### Virulence Assay of Wild Type and *xcc6012* Knockout Mutants

Cabbage (*Brassica oleracea L.* var. capitata L.) was used as the host plant for the leaf clipping assay [37] infected by *Xc*17. Wild type and *xcc6012* knockout mutants were first cultured in the Luria Broth medium until an OD_600_ of 1 was achieved. These mutants were then harvested by centrifugation, and the precipitated cells were further suspended in water to an OD_600_ of 0.001. This suspension was inoculated onto the cut edges of leaves from a potted cabbage plant. Both water and Luria Broth medium were also introduced into the cabbage vascular system at separate clipping sites as negative controls.

### Biophysical Measurements of Wild Type and XCC6012 Variants

*Size-Exclusion Chromatography at 4°C:* 100-µl of wild type and mutated XCC6012 at a concentration of 0.13 mg/ml (approximately 6.4 µM per protein monomer) were applied to a Superdex 75 chromatography column (GE Healthcare) with a flow rate of 0.4 mL/min equilibrated with the lysis buffer (20 mM Tris-HCl, pH 8.0, 80 mM NaCl). Eluted proteins were detected by UV absorption at 280 nm. Molecular weight standards of known Stokes radii (GE Healthcare) of Blue-dextran 2000, albumin, ovalbumin, chymotrypsinogen A and ribonuclease A were used to calibrate the molecular weight of wild type XCC6012 and its variants.

#### Differential Scanning Calorimetry Experiment

The thermo-stability of XCC6012 and its variants were determined by measuring the midpoint melting temperature (T_m_) from the DSC experiment [63] on a N-DSC III device (TA Instrument). Sample cell (300 µL) containing a protein solution of 0.79 mg/mL (approximately 38 µM per protein monomer) was heated at a rate of 1°C/min from 20°C to 90°C. The molar heat capacity versus temperature was computed from the raw data after subtraction of the buffer background by using the attached program NanoAnalyze Data Analysis version 2.0 program (TA Instrument).

#### Isothermal Titration Calorimetry (ITC) Experiment

Each protein sample was dialyzed with a buffer containing 20 mM Tris-HCl (pH 8.0), 80 mM NaCl, 5 mM MgCl_2_, and 10 mM KCl. C-di-GMP powder was dissolved in the same buffer. Measurements were performed using an iTC200 (MicroCal Inc, GE Healthcare) at 25°C with 50 µM XCC6012 protein in a 200-µl sample cell titrated with 3125 µM c-di-GMP. An initial 1 µl injection was followed by 17 injections of 2 µl c-di-GMP, with each injection spaced in 150 s intervals. Binding isotherms were calculated in ORIGIN 7 software (MicroCal Inc, GE Healthcare).

#### Analytical Ultracentrifugation (AUC) Experiments

The sedimentation velocity experiments were performed using a Beckman model XL-A analytical ultracentrifuge. Protein samples and reference solution, which were dialyzed against a buffer solution containing 20 mM Tris-HCl and 80 mM NaCl at pH 8.0, were separately loaded into a conventional double-sector quartz cell and mounted in a Beckman An-50 Ti rotor. Experiments were conducted at 20°C with a rotor speed of 42,000 r.p.m. (130,000 g). Absorbance of the sample at 280 nm was monitored in a continuous mode, with a time interval of 480 s, and a step size of 0.002 cm. Multiple A280 scans (52 for the XCC6012 wild type sample, and 44 for the V71M and K77D variant samples, respectively) at different time points were fitted to a continuous size distribution using the program SEDFIT [44]. The partial specific volume of the protein was set at the default value (0.73 cm^3^ g^−1^) for calculation with the SEDFIT program, and the buffer parameters were estimated by using the Sednterp program [64]. The values chosen for buffer density ρ and viscosity η were 1.002 g cm^−3^ and 0.01015 g cm^−1^ s^−1^, respectively. The initial frictional ratio f/f0 for fitting is set at the default value of 1.2 in the SEDFIT program for calculation. The frictional ratio is also one of the variables for the c(s) curve fitting, which converges to a weight-average value of 1.24 after fitting with the wild type protein, 1.23 with the V71M variant, and 1.27 with the K77D variant, respectively. Normalized size distributions were solved at a confidence level of P = 0.95.

## Supporting Information

Figure S1 **a**)** DNA sequence alignment of** ***xcc6012*** **gene with other Xanthomonas homologues using Clustal W2.** Unmatched nucleotides between sequences are highlighted in light purple. The coding regions for *xcc2249*, *xac1971*, *xoo2585*, and *xcv2018* are downloaded from the website (NC_007086.1∶2708426..2708782, NC_003919.1∶2302787..2303353, NC_006834.1∶2736610..2737176, and NC_007508.1∶2299681..2300247, respectively). However, the coding region of *xc2249* was interpreted begining from codon 71 (boxed in red). Since this codon should code for a Val, not a Met, the notion that the *xc2249* gene starts from codon 71 is likely to be a misannotation in the website. When 210 nucleotides from the upstream region of codon 71 are included, we found that it starts with an initiation ATG codon, similar to other homologous sequences. Translation of this new 567 base-long sequence “NC_007086.1∶2708216..2708782” (including the upstream 210 bases) shall generate a protein length of 188 amino acids, similar to other homologous protein sequences within the Xanthomonads (Fig. S1b). The *xcc6012* and the new *xc2249* DNA sequences are now completely identical (boxed in blue). **b**). **Protein sequence alignment of XCC6012 with homologues from** ***Xanthomonads*** **using Clustal W2**. Unmatched amino acid residues are highlighted in yellow. Residue 71 of XC2249 (GenBank access number: **AAY49304.1**) has been corrected from a Met to a Val (boxed in red). XCC6012 displays high sequence identity with XAC1971 (GenBank access number: **AAM36833.1)** from *Xanthomonas axonopodis* pv. *citri* str. 306, XOO2585 (GenBank access number: **AAW75839.1**) from *Xanthomonas oryzae* pv. *oryzae* KACC10331, and XCV2018 (GenBank access number: **CAJ23695.1**) from *Xanthomonas campestris* pv. *vesicatoria* str. 85-10). The sequence of XCC6012 is completely identical to that of the corrected XC2249 (boxed in blue), and the sequence identity and similarity with those of the XAC1971, XOO2585, and XCV2018 sequences are 79.3%, 79.3%, 79.8% and 87.8%, 87.2%, 88.8%, respectively. **c**) **The neighborhood genes of** ***xcc6012*** **in Xcc strain 17,** ***xc2249*** **in Xcc8004,** ***xac1971*** **in Xac306,** ***xoo2585*** **in Xoo ATCC10331, and** ***xcv2018*** **in Xcv85-10.** The *pilZ* gene is highlighted in cyan, and its upstream unknown gene is highlighted in blue. The further upstream genes coding for *fliC*, *fliD*, and *fliS* are highlighted in green, while the four downstream genes coding for the LuxR-like response regulator, *rpoN*, putative two-component response regulator, and *fleQ* are highlighted in yellow. The top arrows indicate the neighborhood gene order around the *pilZ* gene. It is clear from this figure that the same gene order is found for the *xcc6012* gene in *Xcc* strain 17 as well as in other Xanthomonad species.
(PDF)

Figure S2 **Multiple sequence and structural alignment of the PilZ domains of VCA0042, PA4608 and PP4397 with XCC6012.** The extra α2-α3 helices of XCC6012 were excluded for ease of comparison. The highly conserved RxxxR residues residing in the α1 and loop region (bracketed in blue) and the DxSxxG residues at the β2-β3 loop-turn region are highlighted in red. However, only partial conserved residues of SxxG were identified in XCC6012. The sequence identities of XCC6012 with these PilZ domains are very low, ranging from 13% to 19%.
(PDF)

Figure S3 **Superimposition of XCC6012 (in red) with the apo-form (in blue) and the c-di-GMP complexed form (in green) of VCA0042 drawn in stereo.** a) Binding of c-di-GMP (represented with van der Waals sphere) causes a significant conformational change of the YcgR domain toward the PilZ domain (indicated by a curved light-blue arrow) [7]. The superimposed PilZ domains are boxed in red and shown expanded in the figure below. b) The β3-β6 strands of the PilZ domains can be well superimposed, while the bottom of the β1-strand (marked by blue arrow) and the top of the β2-strand (marked by red and green dotted lines) of XCC6012 diverge to a greater extent to other PilZ domains, due to the insertion of additional α2 and α3 helices between the β1-β2 strands.
(PDF)

Figure S4 **Type III interactions resulting from the outer surface residue of α2 with the inner surface residues of α4, shown in two different orientations.** a) Hydrophobic residues from helix α2 are drawn in light-green spheres while hydrophobic residues from helix α4 are drawn in white spheres. b) The 180° rotational view of Fig. S4a showing the electrostatic interactions between the Asp167 and Arg47/Gly164 residues.
(PDF)

Figure S5 **Titrations of XCC6012 with c-di-GMP using ITC.** The experiments were carried out at 25°C, and the more concentrated c-di-GMP (3125 µM) was titrated into the XCC6012 protein solution (50 µM). An initial 1 µl injection was followed by seventeen 2 µl injections spaced at a 150 s intervals. A binding isotherm curve could not be built, due to the weak binding heat release despite a high protein/c-di-GMP molar ratio.
(PDF)

Figure S6 **The B-factor and electrostatic plots of the XCC6012 tetramer in stereo.** a) The stereo picture of XCC6012 drawn in B-factor. The thicker columns indicates regions with higher B-factors. b) The stereo picture of XCC6012 drawn in electrostatic plot. The positively charged regions are drawn in blue and the negatively charged regions in red. The four clustered positively charged regions are boxed in green.
(PDF)

Figure S7 **Sedimentation velocity data of wild type XCC6012 from absorbance at 280 nm at two different protein concentrations via analytical ultracentrifugation experiments.** (a,d) Sedimentation velocity profiles for XCC6012 at 38.4****µM (a) and 6.4 µM (d) concentration. The data were obtained at different times up to 8 h of sedimentation at 42,000 r.p.m. (130,000 g) at 20°C (every fifth trace was shown). (b,e) Superposition of the difference between the experimental and fitted curves. (c,f) C(s) distribution analysis by the SEDFIT program (http://www.analyticalultracentrifugation.com). (g) Superimposition of the two c(s) distributions for XCC6012 at 38.4 µM (black) and 6.4 µM (green) concentrations. It is obvious that XCC6012 forms a tetramer even at such a low protein concentration, with only a single species of 84 kDa being observed.
(PDF)

Figure S8 **Sedimentation velocity experiments of V71M and K77D variants of the XCC6012 from absorbance data at 280 nm at 38.4 µM concentrations.** (a,d) Sedimentation velocity profiles for V71M (a) and K77D (d). These data were obtained under similar conditions to the wild type XCC6012. (b,e) Superposition of the difference between the experimental and fitted curves. (c,f) C(s) distribution analysis by the Sedfit program. (g) Superimposition of the three c(s) distributions for wild type XCC6012 (black), V71M (blue), and K77D (red) variants. It is obvious that both wild type XCC6012 and V71M variants form a stable tetramer while K77D forms a stable dimer. No other major species could be observed in each c(s) distribution.
(PDF)
