# Supplementary material for: A Novel Tetrameric PilZ Domain Structure from Xanthomonads
Source: PLoS One. 2011 Jul 7;6(7):e22036. doi: 10.1371/journal.pone.0022036 (PMC3131395; doi:10.1371/journal.pone.0022036)
Supplement: Figure S1 — a) DNA sequence alignment of xcc6012 gene with other Xanthomonas homologues using Clustal W2. Unmatched nucleotides between sequences are highlighted in light purple. The coding regions for xcc2249, xac1971, xoo2585, and xcv2018 are downloaded from the website (NC_007086.1∶2708426..2708782, NC_003919.1∶2302787..2303353, NC_006834.1∶2736610..2737176, and NC_007508.1∶2299681..2300247, respectively). However, the coding region of xc2249 was interpreted begining from codon 71 (boxed in red). Since this codon should code for a Val, not a Met, the notion that the xc2249 gene starts from codon 71 is likely to be a misannotation in the website. When 210 nucleotides from the upstream region of codon 71 are included, we found that it starts with an initiation ATG codon, similar to other homologous sequences. Translation of this new 567 base-long sequence “NC_007086.1∶2708216..2708782” (including the upstream 210 bases) shall generate a protein length of 188 amino acids, similar to other homologous protein sequences within the Xanthomonads (Fig. S1b). The xcc6012 and the new xc2249 DNA sequences are now completely identical (boxed in blue). b). Protein sequence alignment of XCC6012 with homologues from Xanthomonads using Clustal W2. Unmatched amino acid residues are highlighted in yellow. Residue 71 of XC2249 (GenBank access number: AAY49304.1) has been corrected from a Met to a Val (boxed in red). XCC6012 displays high sequence identity with XAC1971 (GenBank access number: AAM36833.1) from Xanthomonas axonopodis pv. citri str. 306, XOO2585 (GenBank access number: AAW75839.1) from Xanthomonas oryzae pv. oryzae KACC10331, and XCV2018 (GenBank access number: CAJ23695.1) from Xanthomonas campestris pv. vesicatoria str. 85-10). The sequence of XCC6012 is completely identical to that of the corrected XC2249 (boxed in blue), and the sequence identity and similarity with those of the XAC1971, XOO2585, and XCV2018 sequences are 79.3%, 79.3%, 79.8% and 87.8%, 87.2%, 88.8%, respectively [file pone.0022036.s001.pdf]

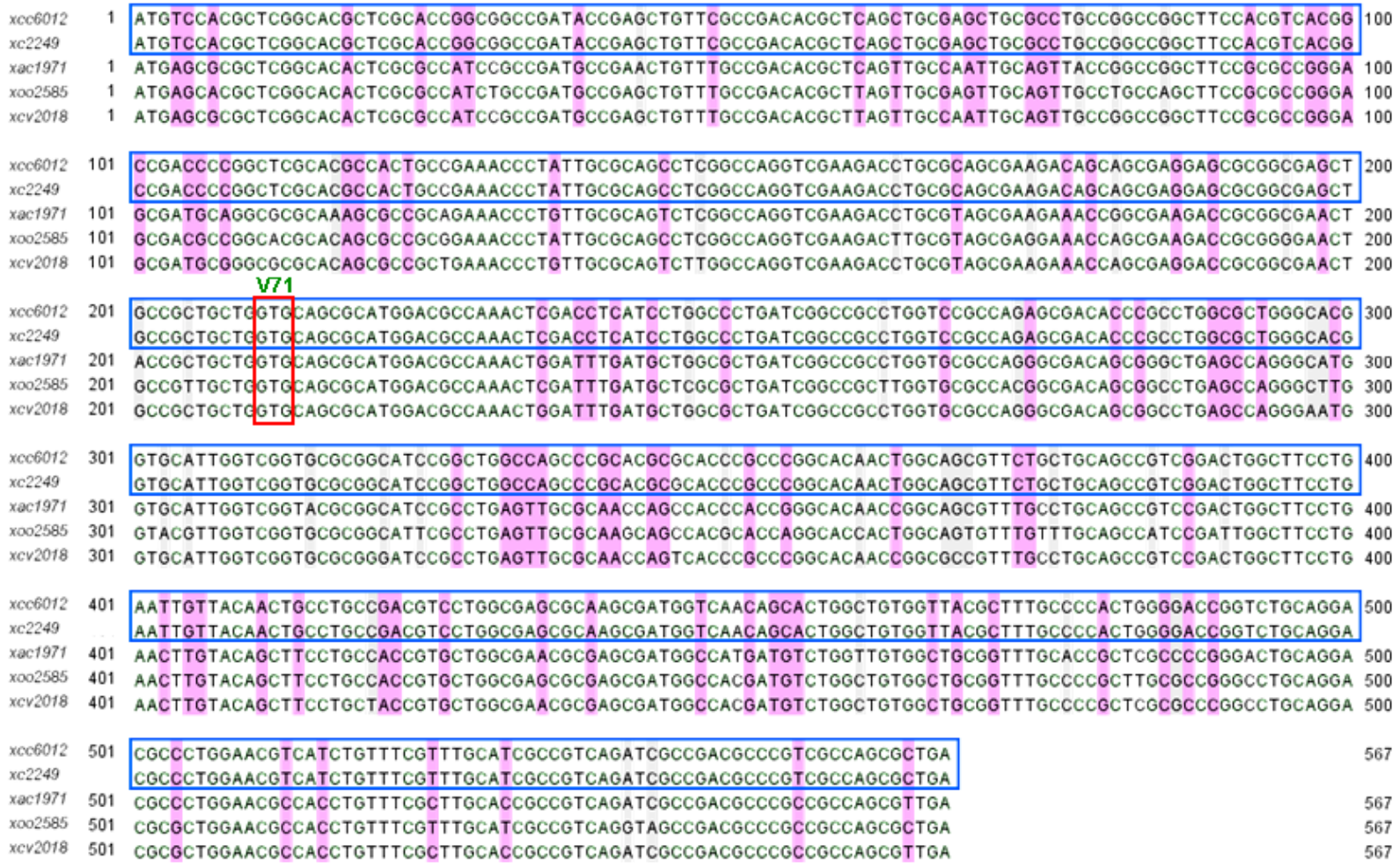
xcc6012 1 ATGTCCACGCTCGGCACGCTCGCACCGGCGGCCGATACCGAGCTGTTCGCCGACACGCTCAGCTGCGAGCTGCGCCTGCCGGCCGGCTTCCACGTCACGG 100
xc2249 ATGTCCACGCTCGGCACGCTCGCACCGGCGGCCGATACCGAGCTGTTCGCCGACACGCTCAGCTGCGAGCTGCGCCTGCCGGCCGGCTTCCACGTCACGG
xac1971 1 ATGAGCGCGCTCGGCACACTCGCGCCATCCGCCGATGCCGAACTGTTTGCCGACACGCTCAGTTGCCAATTGCAGTTACCGGCCGGCTTCCGCGCCGGGA 100
xoo2585 1 ATGAGCACGCTCGGCACACTCGCGCCATCTGCCGATGCCGAGCTGTTTGCCGACACGCTTAGTTGCGAGTTGCAGTTGCCTGCCAGCTTCCGCGCCGGGA 100
xcv2018 1 ATGAGCGCGCTCGGCACACTCGCGCCATCCGCCGATGCCGAGCTGTTTGCCGACACGCTTAGTTGCCAATTGCAGTTGCCGGCCGGCTTCCGCGCCGGGA 100
xcc6012 101 CCGACCCCGGCTCGCACGCCACTGCCGAAACCCTATTGCGCAGCCTCGGCCAGGTCGAAGACCTGCGCAGCGAAGACAGCAGCGAGGAGCGCGGCGAGCT 200
xc2249 CCGACCCCGGCTCGCACGCCACTGCCGAAACCCTATTGCGCAGCCTCGGCCAGGTCGAAGACCTGCGCAGCGAAGACAGCAGCGAGGAGCGCGGCGAGCT
xac1971 101 GCGATGCAGGCGCGCAAAGCGCCGCAGAAACCCTGTTGCGCAGTCTCGGCCAGGTCGAAGACCTGCGTAGCGAAGAAACCGGCGAAGACCGCGGCGAACT 200
xoo2585 101 GCGACGCCGGCACGCACAGCGCCGCGGAAACCCTATTGCGCAGCCTCGGCCAGGTCGAAGACTTGCGTAGCGAGGAAACCAGCGAAGACCGCGGGGAACT 200
xcv2018 101 GCGATGCGGGCGCGCACAGCGCCGCTGAAACCCTGTTGCGCAGTCTTGGCCAGGTCGAAGACCTGCGTAGCGAAGAAACCAGCGAGGACCGCGGCGAACT 200
V71
xcc6012 201 GCCGCTGCTGGTGCAGCGCATGGACGCCAAACTCGACCTCATCCTGGCCCTGATCGGCCGCCTGGTCCGCCAGAGCGACACCCGCCTGGCGCTGGGCACG 300
xc2249 GCCGCTGCTGGTGCAGCGCATGGACGCCAAACTCGACCTCATCCTGGCCCTGATCGGCCGCCTGGTCCGCCAGAGCGACACCCGCCTGGCGCTGGGCACG
xac1971 201 ACCGCTGCTGGTGCAGCGCATGGACGCCAAACTGGATTTGATGCTGGCGCTGATCGGCCGCCTGGTGCGCCAGGGCGACAGCGGGCTGAGCCAGGGCATG 300
xoo2585 201 GCCGTTGCTGGTGCAGCGCATGGACGCCAAACTCGATTTGATGCTCGCGCTGATCGGCCGCTTGGTGCGCCACGGCGACAGCGGCCTGAGCCAGGGCTTG 300
xcv2018 201 GCCGCTGCTGGTGCAGCGCATGGACGCCAAACTGGATTTGATGCTGGCGCTGATCGGCCGCCTGGTGCGCCAGGGCGACAGCGGCCTGAGCCAGGGAATG 300
xcc6012 301 GTGCATTGGTCGGTGCGCGGCATCCGGCTGGCCAGCCCGCACGCGCACCCGCCCGGCACAACTGGCAGCGTTCTGCTGCAGCCGTCGGACTGGCTTCCTG 400
xc2249 GTGCATTGGTCGGTGCGCGGCATCCGGCTGGCCAGCCCGCACGCGCACCCGCCCGGCACAACTGGCAGCGTTCTGCTGCAGCCGTCGGACTGGCTTCCTG
xac1971 301 GTGCATTGGTCGGTACGCGGCATCCGCCTGAGTTGCGCAACCAGCCACCCACCGGGCACAACCGGCAGCGTTTGCCTGCAGCCGTCCGACTGGCTTCCTG 400
xoo2585 301 GTACGTTGGTCGGTGCGCGGCATTCGCCTGAGTTGCGCAAGCAGCCACGCACCAGGCACCACTGGCAGTGTTTGTTTGCAGCCATCCGATTGGCTTCCTG 400
xcv2018 301 GTGCATTGGTCGGTGCGCGGGATCCGCCTGAGTTGCGCAACCAGTCACCCGCCCGGCACAACCGGCGCCGTTTGCCTGCAGCCGTCCGACTGGCTTCCTG 400
xcc6012 401 AATTGTTACAACTGCCTGCCGACGTCCTGGCGAGCGCAAGCGATGGTCAACAGCACTGGCTGTGGTTACGCTTTGCCCCACTGGGGACCGGTCTGCAGGA 500
xc2249 AATTGTTACAACTGCCTGCCGACGTCCTGGCGAGCGCAAGCGATGGTCAACAGCACTGGCTGTGGTTACGCTTTGCCCCACTGGGGACCGGTCTGCAGGA
xac1971 401 AACTTGTACAGCTTCCTGCCACCGTGCTGGCGAACGCGAGCGATGGCCATGATGTCTGGTTGTGGCTGCGGTTTGCACCGCTCGCCCCGGGACTGCAGGA 500
xoo2585 401 AACTTGTACAGCTTCCTGCCACCGTGCTGGCGAGCGCGAGCGATGGCCACGATGTCTGGCTGTGGCTGCGGTTTGCCCCGCTTGCGCCGGGCCTGCAGGA 500
xcv2018 401 AACTTGTACAGCTTCCTGCTACCGTGCTGGCGAACGCGAGCGATGGCCACGATGTCTGGCTGTGGCTGCGGTTTGCCCCGCTCGCGCCCGGCCTGCAGGA 500
xcc6012 501 CGCCCTGGAACGTCATCTGTTTCGTTTGCATCGCCGTCAGATCGCCGACGCCCGTCGCCAGCGCTGA 567
xc2249 CGCCCTGGAACGTCATCTGTTTCGTTTGCATCGCCGTCAGATCGCCGACGCCCGTCGCCAGCGCTGA
xac1971 501 CGCCCTGGAACGCCACCTGTTTCGCTTGCACCGCCGTCAGATCGCCGACGCCCGCCGCCAGCGTTGA 567
xoo2585 501 CGCGCTGGAACGCCACCTGTTTCGTTTGCATCGCCGTCAGGTAGCCGACGCCCGCCGCCAGCGCTGA 567
xcv2018 501 CGCGCTGGAACGCCACCTGTTTCGCTTGCACCGCCGTCAGATCGCCGACGCCCGCCGCCAGCGTTGA 567

Fig. S1a

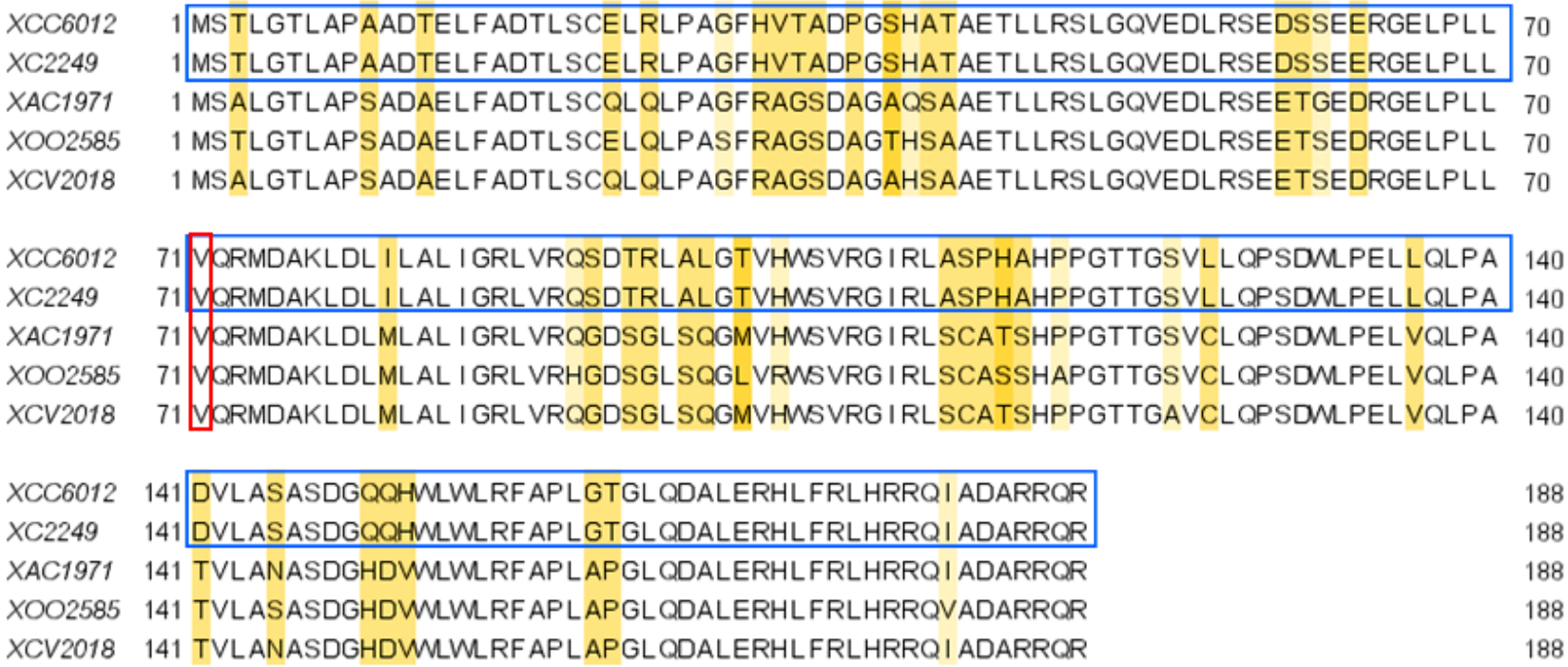
XCC6012 1 MSTLGTLAPAADTELFADTLSCELRLPAGFHVTADPGSHATAETLLRSLGQVEDLRSEDSSEERGELPLL 70
XC2249 1 MSTLGTLAPAADTELFADTLSCELRLPAGFHVTADPGSHATAETLLRSLGQVEDLRSEDSSEERGELPLL 70
XAC1971 1 MSALGTLAPSADAELFADTLSCQLQLPAGFRAGSDAGAQSAAETLLRSLGQVEDLRSEETGEDRGELPLL 70
XOO2585 1 MSTLGTLAPSADAELFADTLSCELQLPASFRAGSDAGTHSAAETLLRSLGQVEDLRSEETSEDRGELPLL 70
XCV2018 1 MSALGTLAPSADAELFADTLSCQLQLPAGFRAGSDAGAHSAAETLLRSLGQVEDLRSEETSEDRGELPLL 70
XCC6012 71 VQRMDAKLDLILALIGRLVRQSDTRLALGTVHWSVRGIRLASPHAHPPGTTGSVLLQPSDWLPELLQLPA 140
XC2249 71 VQRMDAKLDLILALIGRLVRQSDTRLALGTVHWSVRGIRLASPHAHPPGTTGSVLLQPSDWLPELLQLPA 140
XAC1971 71 VQRMDAKLDLMLALIGRLVRQGDSGLSQGMVHWSVRGIRLSCATSHPPGTTGSVCLQPSDWLPELVQLPA 140
XOO2585 71 VQRMDAKLDLMLALIGRLVRHGDSGLSQGLVRWSVRGIRLSCASSHAPGTTGSVCLQPSDWLPELVQLPA 140
XCV2018 71 VQRMDAKLDLMLALIGRLVRQGDSGLSQGMVHWSVRGIRLSCATSHPPGTTGAVCLQPSDWLPELVQLPA 140
XCC6012 141 DVLASASDGQQHWLWLRFAPLGTGLQDALERHLFRLHRRQIADARRQR 188
XC2249 141 DVLASASDGQQHWLWLRFAPLGTGLQDALERHLFRLHRRQIADARRQR 188
XAC1971 141 TVLANASDGHDWWLWLRFAPLAPGLQDALERHLFRLHRRQIADARRQR 188
XOO2585 141 TVLASASDGHDWWLWLRFAPLAPGLQDALERHLFRLHRRQVADARRQR 188
XCV2018 141 TVLANASDGHDWWLWLRFAPLAPGLQDALERHLFRLHRRQIADARRQR 188

Fig. S1b

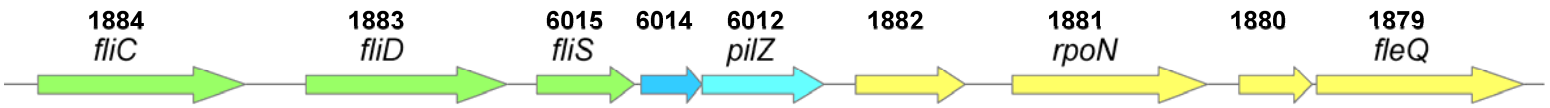

Gene name	Gene product	Gene number in *Xcc17*	Gene number in *Xcc8004*	Gene number in *Xac306*	Gene number in *Xoo ATCC10331*	Gene number in *Xcv 85-10*
fliC	flagellin	XCC1884	XC2245	XAC1975	XOO2581	XCV2022
fliD	flagellar capping protein	XCC1883	XC2246	XAC1974	XOO2582	XCV2021
fliS	flagellin-specific chaperone FliS	XCC6015	XC2247	XAC1973	XOO2583	XCV2020
	hypothetical protein	XCC6014	XC2248	XAC1972	XOO2584	XCV2019
pilZ	XCC6012 homolog	XCC6012	XC2249	XAC1971	XOO2585	XCV2018
	LuxR family two-component response regulator	XCC1882	XC2250	XAC1970	XOO2586	XCV2017
rpoN	RNA polymerase σ-54 factor	XCC1881	XC2251	XAC1969	XOO2587	XCV2016
	putative two-component response regulator	XCC1880	XC2252	XAC1968	XOO2588	XCV2015
fleQ	σ-54 dependent transcriptional activator FleQ	XCC1879	XC2253	XAC1967	XOO2589	XCV2014

Fig. S1c
