# Supplementary figures and images for: A Novel Tetrameric PilZ Domain Structure from Xanthomonads
Source: PLoS One. 2011 Jul 7;6(7):e22036. doi: 10.1371/journal.pone.0022036 (PMC3131395; doi:10.1371/journal.pone.0022036)

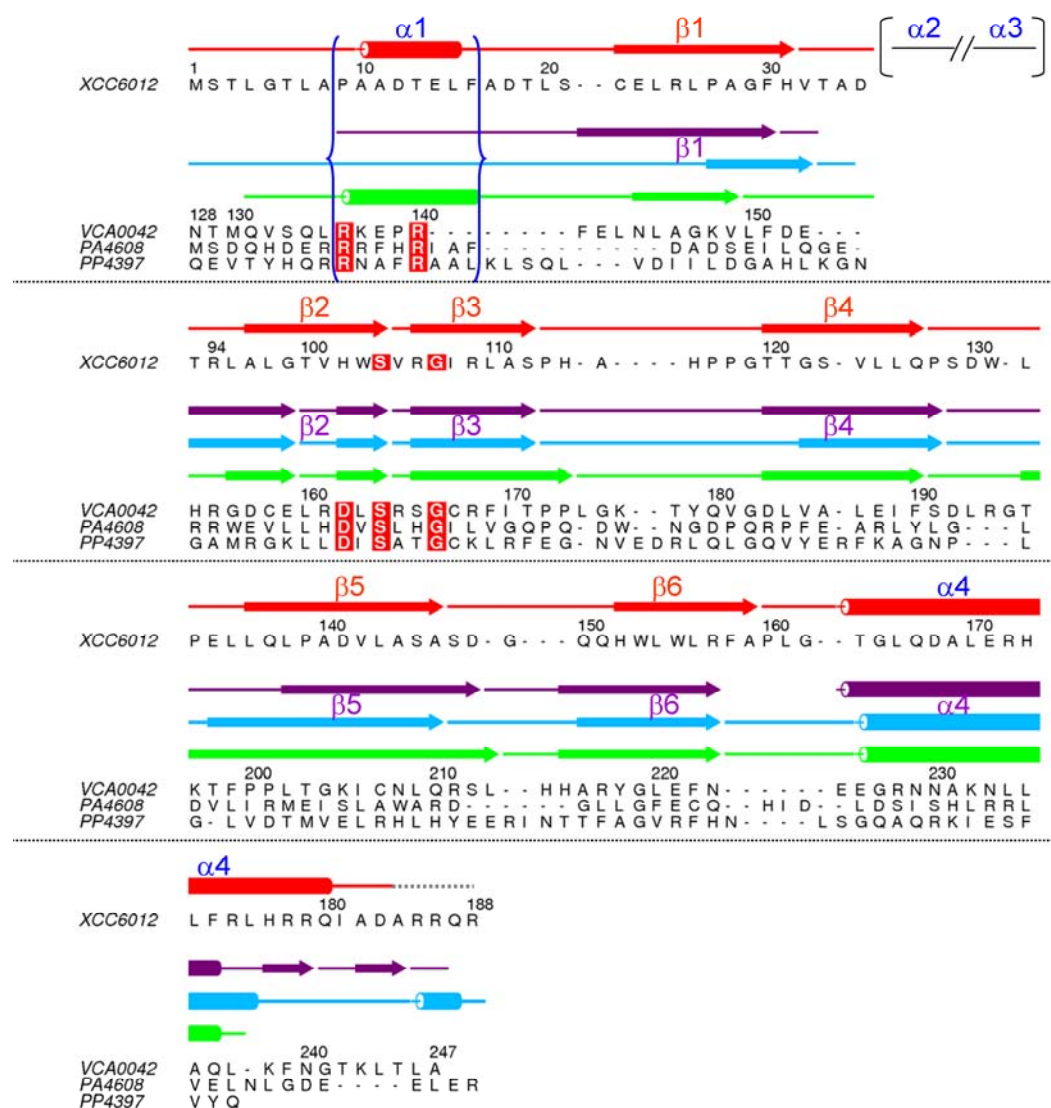

Fig. S2

Supplement: Figure S2 — Multiple sequence and structural alignment of the PilZ domains of VCA0042, PA4608 and PP4397 with XCC6012. The extra α2-α3 helices of XCC6012 were excluded for ease of comparison. The highly conserved RxxxR residues residing in the α1 and loop region (bracketed in blue) and the DxSxxG residues at the β2-β3 loop-turn region are highlighted in red. However, only partial conserved residues of SxxG were identified in XCC6012. The sequence identities of XCC6012 with these PilZ domains are very low, ranging from 13% to 19%. (PDF) [file pone.0022036.s002.pdf]

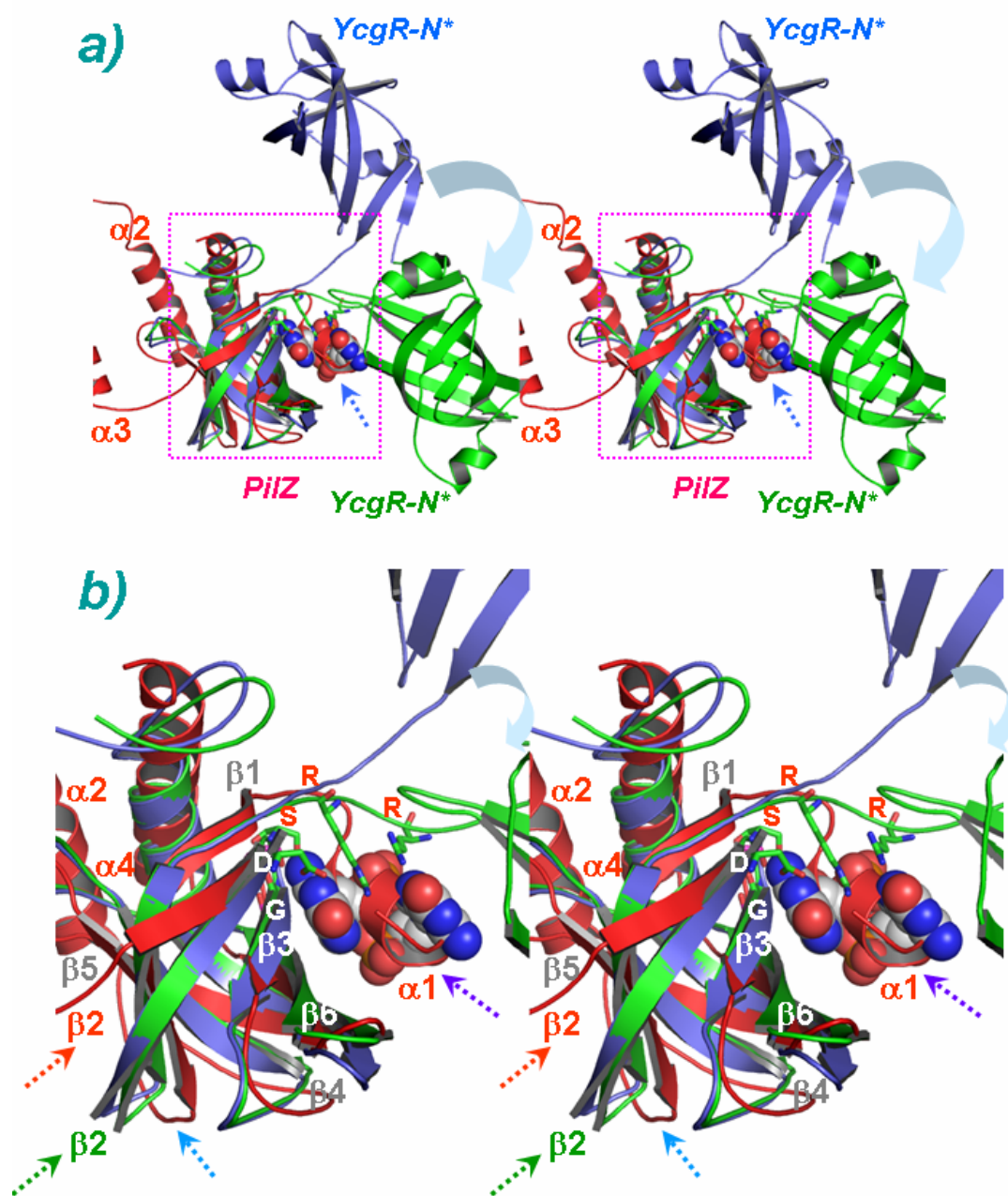

Fig. S3

Supplement: Figure S3 — Superimposition of XCC6012 (in red) with the apo-form (in blue) and the c-di-GMP complexed form (in green) of VCA0042 drawn in stereo. a) Binding of c-di-GMP (represented with van der Waals sphere) causes a significant conformational change of the YcgR domain toward the PilZ domain (indicated by a curved light-blue arrow) [7]. The superimposed PilZ domains are boxed in red and shown expanded in the figure below. b) The β3-β6 strands of the PilZ domains can be well superimposed, while the bottom of the β1-strand (marked by blue arrow) and the top of the β2-strand (marked by red and green dotted lines) of XCC6012 diverge to a greater extent to other PilZ domains, due to the insertion of additional α2 and α3 helices between the β1-β2 strands. (PDF) [file pone.0022036.s003.pdf]

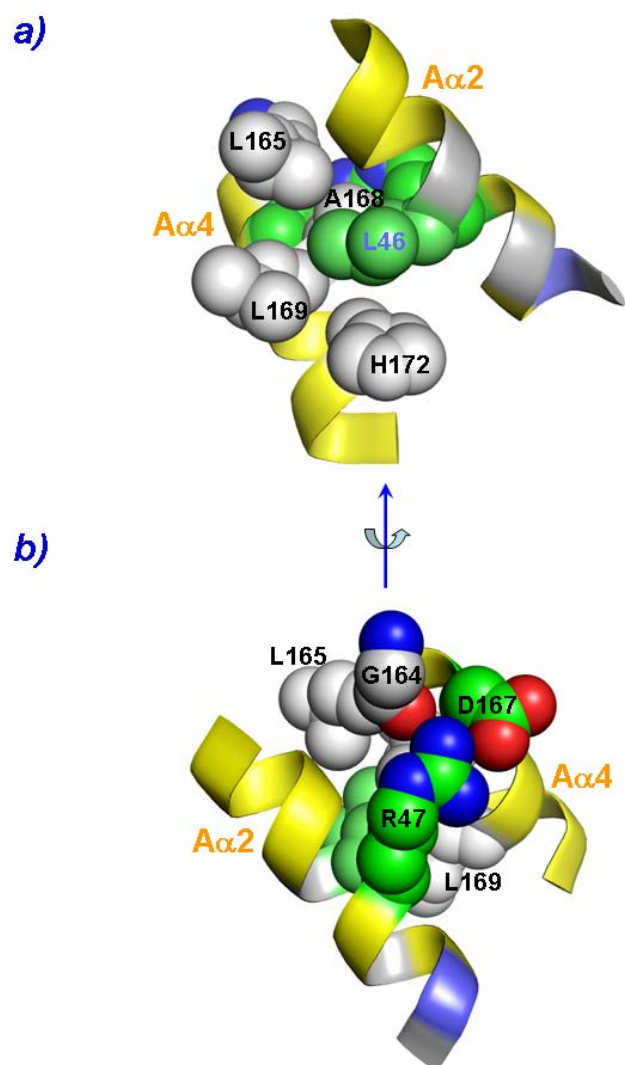

Fig. S4

Supplement: Figure S4 — Type III interactions resulting from the outer surface residue of α2 with the inner surface residues of α4, shown in two different orientations. a) Hydrophobic residues from helix α2 are drawn in light-green spheres while hydrophobic residues from helix α4 are drawn in white spheres. b) The 180° rotational view of Fig. S4a showing the electrostatic interactions between the Asp167 and Arg47/Gly164 residues. (PDF) [file pone.0022036.s004.pdf]

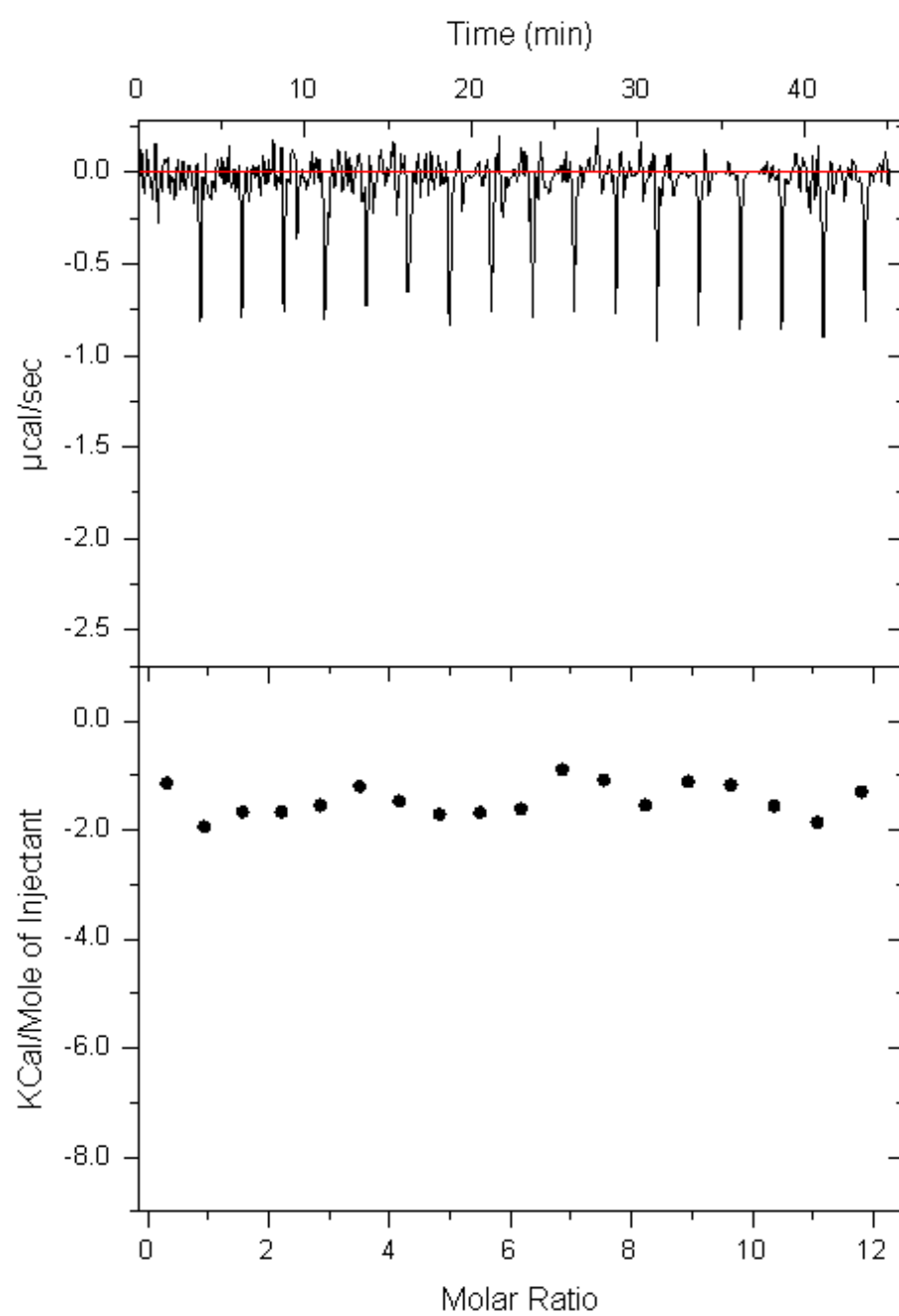

Fig. S5

Supplement: Figure S5 — Titrations of XCC6012 with c-di-GMP using ITC. The experiments were carried out at 25°C, and the more concentrated c-di-GMP (3125 µM) was titrated into the XCC6012 protein solution (50 µM). An initial 1 µl injection was followed by seventeen 2 µl injections spaced at a 150 s intervals. A binding isotherm curve could not be built, due to the weak binding heat release despite a high protein/c-di-GMP molar ratio. (PDF) [file pone.0022036.s005.pdf]

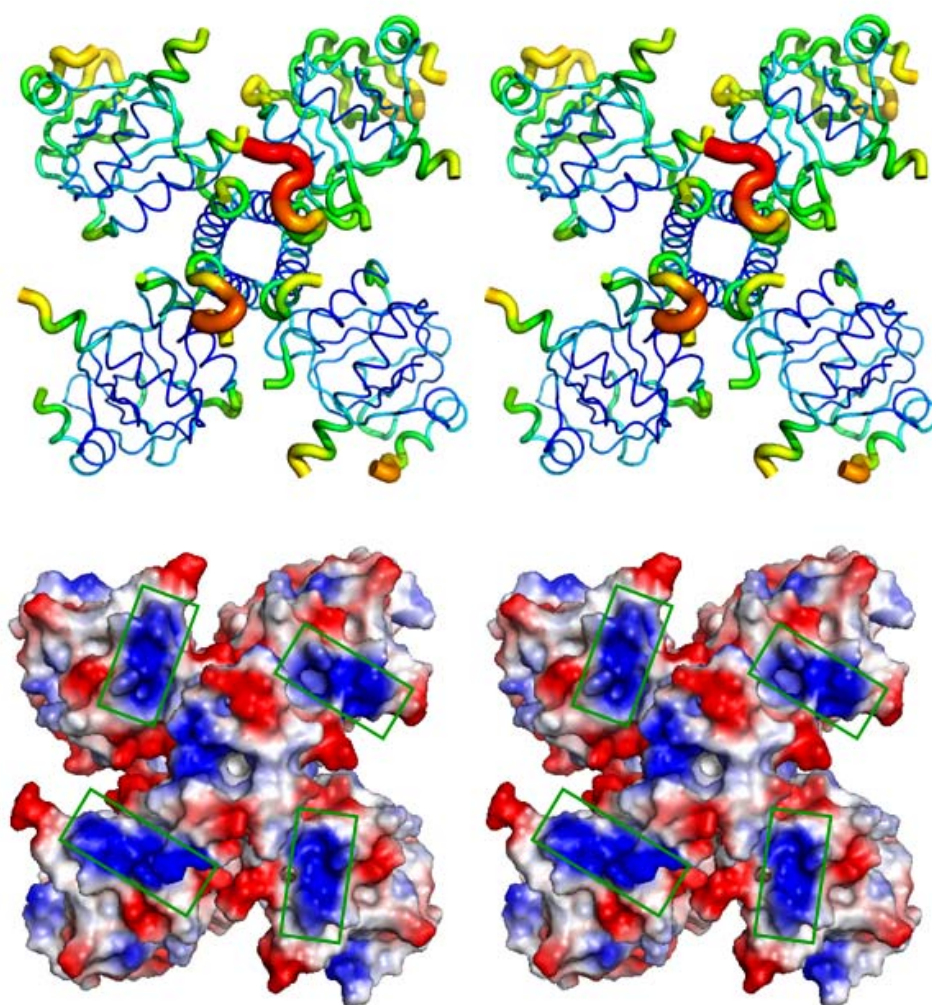

Fig. S6

Supplement: Figure S6 — The B-factor and electrostatic plots of the XCC6012 tetramer in stereo. a) The stereo picture of XCC6012 drawn in B-factor. The thicker columns indicates regions with higher B-factors. b) The stereo picture of XCC6012 drawn in electrostatic plot. The positively charged regions are drawn in blue and the negatively charged regions in red. The four clustered positively charged regions are boxed in green. (PDF) [file pone.0022036.s006.pdf]

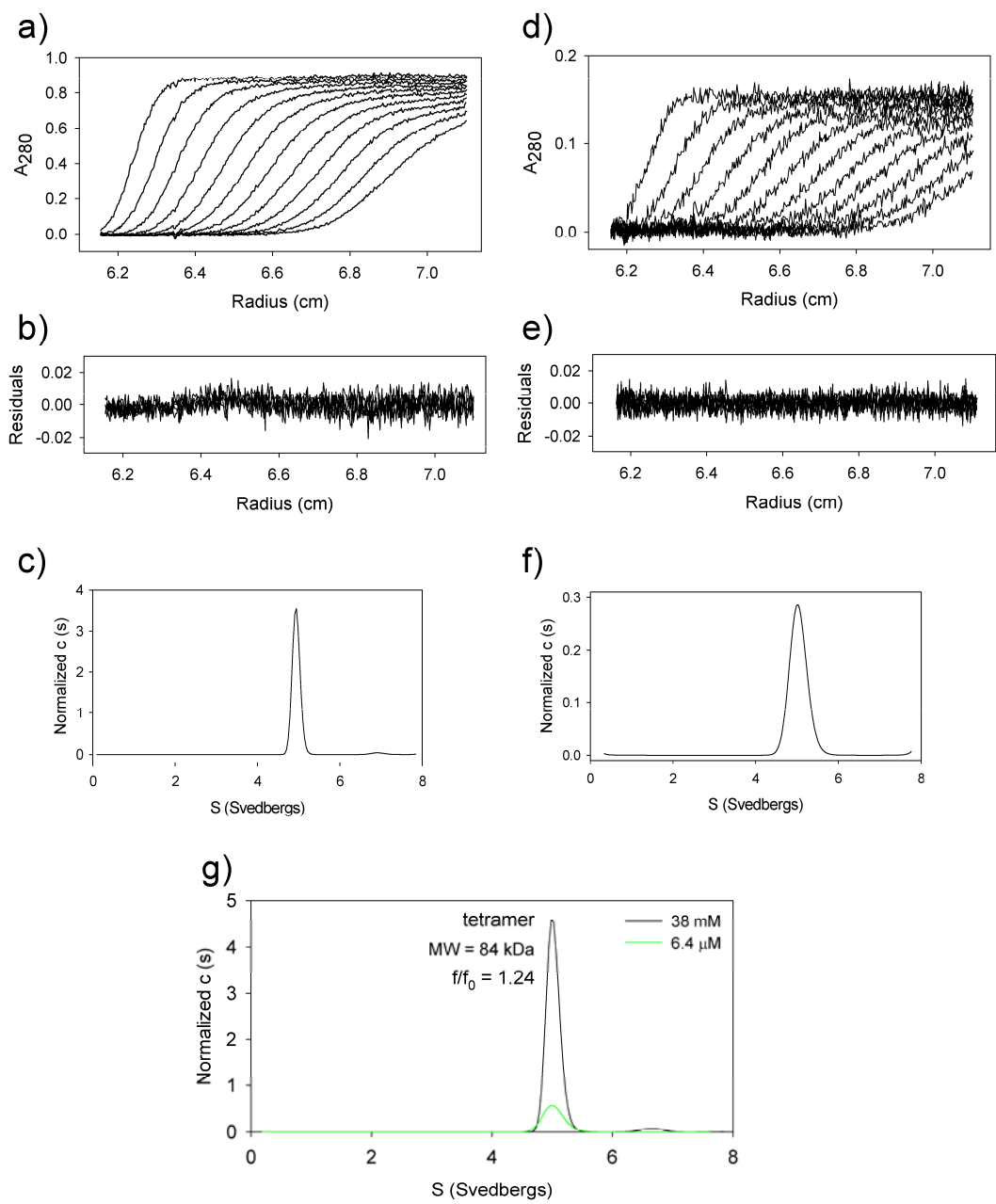

Fig. S7

Supplement: Figure S7 — Sedimentation velocity data of wild type XCC6012 from absorbance at 280 nm at two different protein concentrations via analytical ultracentrifugation experiments. (a,d) Sedimentation velocity profiles for XCC6012 at 38.4µM (a) and 6.4 µM (d) concentration. The data were obtained at different times up to 8 h of sedimentation at 42,000 r.p.m. (130,000 g) at 20°C (every fifth trace was shown). (b,e) Superposition of the difference between the experimental and fitted curves. (c,f) C(s) distribution analysis by the SEDFIT program (http://www.analyticalultracentrifugation.com). (g) Superimposition of the two c(s) distributions for XCC6012 at 38.4 µM (black) and 6.4 µM (green) concentrations. It is obvious that XCC6012 forms a tetramer even at such a low protein concentration, with only a single species of 84 kDa being observed. (PDF) [file pone.0022036.s007.pdf]

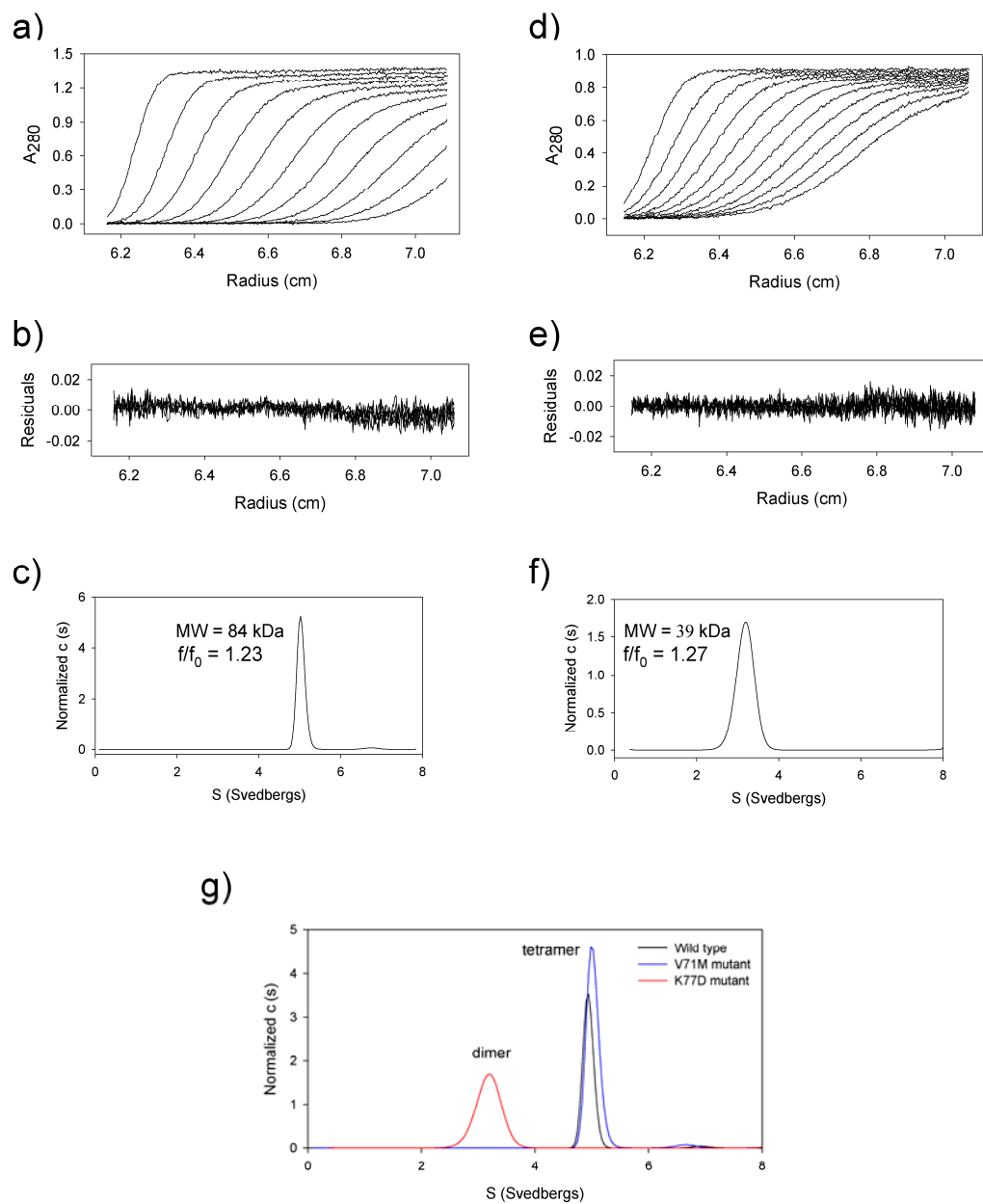

Fig. S8

Supplement: Figure S8 — Sedimentation velocity experiments of V71M and K77D variants of the XCC6012 from absorbance data at 280 nm at 38.4 µM concentrations. (a,d) Sedimentation velocity profiles for V71M (a) and K77D (d). These data were obtained under similar conditions to the wild type XCC6012. (b,e) Superposition of the difference between the experimental and fitted curves. (c,f) C(s) distribution analysis by the Sedfit program. (g) Superimposition of the three c(s) distributions for wild type XCC6012 (black), V71M (blue), and K77D (red) variants. It is obvious that both wild type XCC6012 and V71M variants form a stable tetramer while K77D forms a stable dimer. No other major species could be observed in each c(s) distribution. (PDF) [file pone.0022036.s008.pdf]
